# The Ovarian Development Genes of Bisexual and Parthenogenetic *Haemaphysalis longicornis* Evaluated by Transcriptomics and Proteomics

**DOI:** 10.3389/fvets.2021.783404

**Published:** 2021-12-15

**Authors:** Tianhong Wang, Tongxuan Wang, Meng Zhang, Xinyue Shi, Miao Zhang, Hui Wang, Xiaolong Yang, Zhijun Yu, Jingze Liu

**Affiliations:** ^1^Hebei Key Laboratory of Animal Physiology, Biochemistry and Molecular Biology, College of Life Sciences, Hebei Normal University, Shijiazhuang, China; ^2^Department of Biochemistry and Molecular Biology, College of Basic Medicine, Hebei University of Chinese Medicine, Shijiazhuang, China

**Keywords:** *Haemaphysalis longicornis*, parthenogenetic, ovarian development, transcriptome, proteome, quantitative expression profile

## Abstract

The tick *Haemaphysalis longicornis* has two reproductive groups: a bisexual group (HLBP) and a parthenogenetic group (HLPP). The comparative molecular regulation of ovarian development in these two groups is unexplored. We conducted transcriptome sequencing and quantitative proteomics on the ovaries of HLBP and HLPP, in different feeding stages, to evaluate the molecular function of genes associated with ovarian development. The ovarian tissues of HLBP and HLPP were divided into three feeding stages (early-fed, partially-fed and engorged). A total of 87,233 genes and 2,833 proteins were annotated in the ovary of *H. longicornis* in the different feeding stages. The differentially expressed genes (DEGs) of functional pathway analysis indicated that Lysosome, MAPK Signaling Pathway, Phagosome, Regulation of Actin Cytoskeleton, Endocytosis, Apoptosis, Insulin Signaling Pathway, Oxidative Phosphorylation, and Sphingolipid Metabolism were most abundant in the ovary of *H. longicornis* in the different feeding stages. Comparing the DEGs between HLBP and HLPP revealed that the ABC Transporter, PI3K-Akt Signaling Pathway and cAMP Signaling Pathway were the most enriched and suggested that the functions of signal transduction mechanisms may have changed during ovarian development. The functions of the annotated proteome of ovarian tissues were strongly correlated with the transcriptome annotation results, and these were further validated using quantitative polymerase chain reaction (qPCR). In the HLBP, the expression of cathepsin L, secreted proteins and glycosidase proteins was significantly up-regulated during feeding stages. In the HLPP, the lysozyme, yolk proteins, heat shock protein, glutathione S transferase, myosin and ATP synthase proteins were up-regulated during feeding stages. The significant differences of the gene expression between HLBP and HLPP indicated that variations in the genetic background and molecular function might exist in the two groups. These results provide a foundation for understanding the molecular mechanism and exploring the functions of genes in the ovarian development of different reproductive groups of *H. longicornis*.

**Graphical Abstract:**
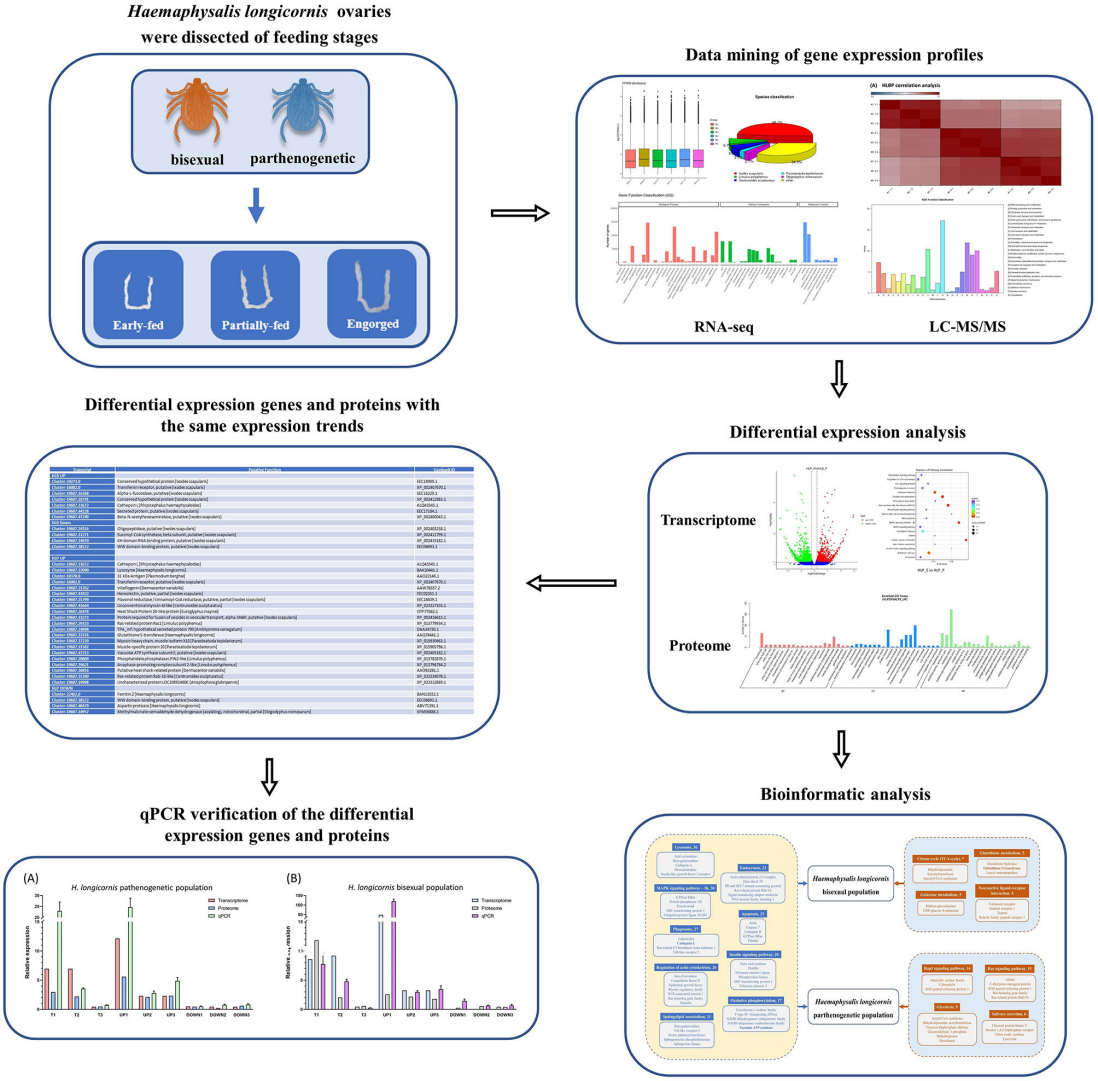
The tick *Haemaphysalis longicornis* has two reproductive groups: a bisexual group (HLBP) and a parthenogenetic group (HLPP). We conducted transcriptome sequencing and quantitative proteomics on the ovaries of HLBP and HLPP, in different feeding stages, to evaluate the molecular function of genes associated with ovarian development. The ovarian tissues of HLBP and HLPP were divided into three feeding stages (early-fed, partially-fed and engorged). The present study discusses genes important for the early development of the tick reproductive system through comparative analysis of differentially expressed genes and proteins at different stages, providing valuable insights into candidate vaccines to control the spread of tick populations.

## Introduction

Ticks are hematophagous ectoparasites of vertebrates (1, 2). A total of 960 tick species have been described worldwide, including species of Ixodidae, Argasidae, Nuttalliellidae, and Deinocrotonidae (3). At least 124 tick species occur in China (4). Many of them are vectors of *Rickettsia, Babesia* and other pathogens (5), and the ticks and resulting tick-borne diseases (TBDs) cause economic losses of billions of US dollars each year (6). During feeding, ticks cause local tissue congestion, inflammation and pathogen transmission to the host. Some pathogens can also be vertically transmitted vertically through eggs (7). Ticks have a strong reproductive capacity, and females begin to lay eggs about 20–30 days after engorgement (8). Each female can lay thousands of eggs (9). The high reproductive capacity can affect population spread as well as the transmission of tick-borne pathogens (4, 10).

*Haemaphysalis longicornis* is a widely distributed tick in New Zealand, Australia and eastern Asia (11, 12). It is an important vector of *Theileria orientalis, Anaplasma phagocytophilum* and *Rickettsia japonica*, and it has spread to 12 states in the USA since it was first identified in 2017 (13). Additionally, there is experimental evidence that this tick is able to transmit *Babesia* spp. that affect domestic livestock. *H. longicornis* has been shown to be the vector of tick-borne encephalitis virus (TBEV) and severe fever with thrombocytopenia syndrome virus (SFTSV) (14). These tick-borne pathogens target domestic and wild animals, often resulting in high mortality rates, which can cause significant damage to livestock economies (15). There are two reproductive groups of *H. longicornis*: the bisexual population (HLBP) and the parthenogenetic population (HLPP). The morphological differences of these two reproductive groups are mainly observed in the female genital pore and Haller's organ (16, 17). HLPP lays fewer eggs than HLBP. However, the engorged body weight and the egg size of HLPP females are greater (18). Cytological studies showed that the chromosome number of HLPP is 30–35, and that of HLBP is 21–22, which may contribute to the reproductive isolation of the two groups (18). Copulation is necessary for the HLBP females to begin engorgement, whereas no males are needed in the HLPP population. HLPP females can complete engorgement and oocyte development without mating (19). Even when HLBP males were available for HLPP females during feeding, no copulation was observed (20).

Parthenogenetic species are usually formed by asexual individuals that mutated during evolutionary adaptation of sexual progenitors; they are unable to undergo gene recombination during reproduction (21). This greatly reduces the evolutionary efficiency of the genes and may cause extinction when environmental conditions change. However, the gene mutation may help rescue the adaptations of the parthenogenetic species (21). Once the parthenogenetic species spreads to a new area, they will be able to effectively utilize the environmental resources and occupy favorable ecological niches (22). In insects, the origins of parthenogenesis mainly include spontaneous origin, hybrid origin, contagious origin and infectious origin (21). The origin of parthenogenetic in ticks is unexplored. Previous studies found differences in chromosomal ploidy, and inferred significant changes in the genetic background of the two groups. Also, it is implied that different ovarian gene functions and regulatory mechanisms between HLPP and HLBP.

The development of the ovaries of *H. longicornis* can be divided into several stages including unfed, slow-feeding, fast feeding, engorgement, pre-oviposition, oviposition onset, and oviposition (21). The ovary in the unfed period is small, and the oocytes are undeveloped. During the slow-feeding period, oocytes develop and ovaries expand. During the fast-feeding period, oocytes gradually accumulate protein precursors and other nutrient molecules. It is clear that oocytes of ticks begin to develop after blood uptake, and the subsequent ovarian development is accompanied by a large number of changes in gene expression. However, knowledge on the molecular regulation of tick ovarian development is meager (23).

In the present study, transcriptomic and proteomic expression profiles of HLBP and HLPP were analyzed in the ovarian tissues of *H. longicornis* at different feeding stages including the early-fed, partially-fed and engorgement stages (The morphological development of the ovaries corresponds to slow-feeding, fast feeding, engorgement) (19). The results will provide new perspective for the understanding of the molecular regulation of parthenogenetic reproduction in ticks, and will provide valuable resources for exploring new targets for subsequent tick control.

## Materials and Methods

### Tick Collection and Feeding

Free-living *H. longicornis* were collected by flag-dragging and placed in a clean, ventilated 50 mL centrifuge tube. HLBP was collected from the Xiaowutai National Nature Reserve Area (40° 03' 03” N, 115° 23' 15” E), Hebei Province, and HLPP was collected from Cangxi County (31° 44' 35” N, 105° 49' 04” E), Sichuan Province, China. Under laboratory conditions, the two populations were fed and reared separately. Briefly, the ticks were put on the ears of the rabbit (Oryctolagus cuniculus) for feeding, and kept in environmental chamber (26 ± 1°C, RH 75%, L: D = 12 h: 12 h) during non-parasitic period. All animal experiments were performed according to the protocols approved by the Animal Care and Use Committee of the Animal Ethics Committee of Hebei Normal University (IACUC-157026).

### Ovarian Dissection and RNA Extraction

Ovaries were dissected from three feeding stages of HLBP and HLPP: early-fed (fed for 48 h), partially-fed (fed for 96 h) and engorgement (detached freely). Each group contained 30 ovaries that were placed in a precooled 1.5 mL centrifuge tube. Precooled 0.01 M PBS (135 mM NaCl, 2.7 mM KCl, 1.5 mM KH_2_PO_4_, 8 mM K_2_HPO_4_, pH 7.4) and 200 mg small-diameter mill beads were used to grind the ovary for 2 min (300 rpm). The samples of ground ovarian tissue in each group were mixed with 1 mL Trizol (Invitrogen, USA), and chilled on ice for 5 min. The sample was mixed with 500 μL chloroform, stirred for 30 s, placed on ice for 2 min, and centrifuged for 15 min (4°C, 12,000 × g). The supernatant containing RNA was pipetted into a new 1.5 mL centrifuge tube. Next, 500 μL precooled isopropanol was added to the sample, gently shaken for 30 s, and placed on ice for 5 min. The sample was centrifuged for 15 min (4°C, 12,000 × g), and the supernatant was discarded. We then added 1 mL 85% ethanol, which was centrifuged for 5 min (4°C, 12,000 × g), and then we discarded the supernatant, and repeated this once. The remaining ethanol was evaporated on an ultra-clean bench for 2 min. RNA purity and integrity were checked by a NanoDrop® 1000 spectrophotometer (IMPLEN, Germany) and a 1% agarose gel.

### Transcriptome Sequencing, Assembly and Annotation

A 1.5 μg RNA sample was purified with poly-T oligonucleotide magnetic beads, and then synthesized by divalent cation in NEB Next® First Strand Synthesis Reaction Buffer (5 ×) (Illumina, USA) at high temperature. First strand cDNA was synthesized by Hexa base random primer and M-MuLV reverse transcriptase. The second strand cDNA was synthesized by DNA polymerase I, dNTPs, and RNase. The Phusion DNA polymerase, universal primer, and Index primer in the AMP XP kit (Beckman Coulter, USA) were used to purify cDNA products, and the data quality was evaluated with an Agilent Bioanalyzer 2100 system (Agilent Technologies, USA). Using the NEB Next® RNA kit (Illumina, USA), the sequence library was generated for paired reads for the Illumina Hiseq platform (Illumina, USA), and paired reads sequencing was performed.

Through CASAVA base recognition analysis, the original image data file was converted to the original sequence, and the results of the sequence were stored in FASTQ file format. The Clean Reads data were assembled and spliced with Trinity software for transcripts data (24). Raw data were deposited with the Sequence Read Archives PRJNA759106 at the National Center for Biotechnology Information (NCBI).

The transcript data were annotated and subject to enrichment analysis. The gene functions in the Gene Ontology (GO) database were divided into Biological process, Molecular function, and Cell function. The Kyoto Encyclopedia of Genes and Genomes (KEGG) database was used to annotate the functional classification of gene products and compounds in the cellular metabolic pathway. Based on the NCBI data, the euKaryotic Ortholog Group (KOG) database annotated the lineal homologous gene analysis, and combined with the evolution relationship, the homologous genes of different species were divided into different homologous families.

### Protein Digestion and Desalination

The ovaries were dissected from three feeding stages of HLBP and HLPP: early-fed (feed for 48 h), partially-fed (feed for 96 h) and engorgement stages (detached freely). The ovaries were placed in a precooled 1.5 mL centrifuge tube. Precooled 0.01 M PBS (135 mM NaCl, 2.7 mM KCl, 1.5 mM KH_2_PO_4_, 8 mM K_2_HPO_4_, pH 7.4) and 200 mg small-diameter mill beads were used to grind the ovary for 2 min (600 rpm). After grinding, the tissue homogenate was transferred to a 15 mL centrifuge tube and centrifuged for 15 min (4°C, 12,000 × g). The supernatant was then transferred to a new 15 mL centrifuge tube, with 1 mL precooled Tris-phenol (Invitrogen, USA) added into the supernatant and vortex oscillated for 1 min. The sample was then centrifuged for 15 min (4°C, 12,000 × g), and the upper aqueous phase was discarded. We added the same volume of 50 mM Tris-HCl (pH = 8.0) (Invitrogen, USA) to the sample, which was then vortex oscillated for 1 min, centrifuged for 15 min (4°C, 12,000 × g), and then the upper aqueous phase was discarded. The supernatant was discarded and five times the volume of 0.1 M precooled ammonium methanol acetate (Invitrogen, USA) was added. The mixed solution was swirled for 1 min and kept at −20°C for about 8 h. Then sample was centrifuged for 15 min (4°C, 12,000 × g) and the supernatant was discarded, after which 0.1 M precooled ammonium methanol acetate was added and the washing steps were repeated one more time. We then added 1 mL −20°C precooled methanol and slowly flushed the sample, transferred it to the new 1.5 mL centrifuge tube, and then centrifuged and discarded the supernatant. We freeze-dried the sample and stored it at −80°C.

The Bradford method was used to determine the protein concentration (25). For each group, the proteins were reduced by adding 0.1 M dithiothreitol (DTT) and maintained at constant temperature of 37°C for 1 h, and then alkylated by adding 0.1 M ioacetamide (IAA) and maintained at constant temperature of 25°C for 30 min. Then, the samlpe was digested with trypsin (Promega, USA) at 37°C for more than 12 h, continually supplemented with enzyme. After the water bath, the enzyme was added to the samlpe again. The products were centrifuged for 15 min (4°C, 14,000 × g), and the supernatant was transferred to a new 15 mL centrifuge tube. The supernatant was desalted using an activated SPE C18 desalting column (Waters, USA). The new syringe used to added 2 mL acetonitrile (10, 20, 30, 40, and 50% concentration) to elute the peptide fragments. The sample was concentrated at low temperature to 100 μL and stored at −80°C.

### LC-MS/MS Analysis

A 20 μL protein sample was combined with 1 μL 0.1% formic acid. All samples were centrifuged and concentrated at low temperature to 100 μL by HPLC (Waters, USA). The sample was divided into 10 components, separated by a C18 column (Waters, USA). The elution procedure was set as: ammonium hydroxide adjusted pH = 10.0, flow rate =1 mL/min, solvent gradient: 2% B = 3 min, 5% B = 19 min, 50% B = 89 min, 90% B = 90 min. Fractions were collected every minute and then merged into 10 separate samples. After low-temperature rotary drying, samples were re-dissolved with 0.1% formic acid water and iRT. Mass spectrometry data were collected using the data-dependent acquisition mode (DDA) of the Q Exactive HF mass spectrometry platform (Thermo Fisher, USA). The enrichment column was a C18 trap column (Agela Technologies, USA). Full scan ranged from 350 to 1,800 m/z, isolation width was 1.5 m/z, and resolution was 60,000. Fragments were obtained by higher-energy collisional dissociation with a normalized collisional energy of 28%. The dynamic exclusion time was set to 30 s. The maximum ion implantation time was set to 50 ms, and the target value of the Automatic Gain Control (AGC) was set to 1 × 10^6^ ions. The dissolution of each component and the HPLC setting procedures were consistent with that of the library method. Mass spectrometry data were collected using the data-independent acquisition mode (DIA) of the Q Exactive HF mass spectrometry platform. The normalized collision energy NCE was set to 27%, the maximum injection time was set to Auto, and the ion implantation model was used for data detection. Three replicates were performed for DIA analysis.

### Protein Identification and Annotation

The Proteome Discoverer software (Thermo Fisher, USA) was used to analyze the DDA data (26). The algorithm used maxLFQ with fixed (cysteine carbamido-methylation) and variable (methionine oxidation) modifications. The polypeptide error rate was 1%. Biognosys Spectronaut software (Biognosys, Switzerland) was used to analyze the expression levels of DIA data (27); the data extraction window was set to Dynamic, the correction factor was set to 1, the identification was set to Normal distribution p-value estimator, and the profiling was set to iRT. The *H. longicornis* ovary transcriptome database was utilized to screen and annotate the proteins. The functional annotation of the proteome data also used the GO, KOG and KEGG databases.

### Transcriptome and Proteome Expression Level Analysis

The transcriptome data was analyzed using RSEM software to quantify gene expression of the Clean Reads assembly results (28). The software used the read count of genes converted to FPKM values to assess the level of gene expression in each set of samples. The DESeq2 method was used to test the independent statistical hypothesis test for DEGs between three replicates with the screening threshold for padj <0.05 and | log2FoldChange |>1 (29). The different gene numbers and expression levels are shown by volcano scatter plots (30). DEGs were extracted for GO functional enrichment analysis and KEGG pathway enrichment analysis, and the histogram scatter plots show the p-values of gene expression level differences (31). Differentially expressed proteins (DEPs) were determined by a fold change difference of log2 > 0.58 or < -0.58 and Q-value <0.05. GO and KEGG enrichment analyses of DEPs used the same methods and parameters as the transcriptome analysis.

### Validation of DEGs and DEPs With Quantitative PCR

The relative expression of the overlap DEGs of the *H. longicornis* ovary in different feeding statuses found by transcriptome and proteome was validated by quantitative polymerase chain reaction (qPCR) using the EasyPure® RNA Kit (TransGen Biotech, China). The gene-specific primers for the validated genes were designed using the sequences from transcriptome database (Supplementary Table S1). The cDNA library was constructed with 500 ng RNA, 10 μL pure water, and TranScript® First-Strand cDNA. The conditions of amplification were: 65°C, 5 min; 2 min ice bath; 42°C, 15 min; and the sample was preserved at −20°C. The primer was designed by Primer premier software (PREMIER Biosoft, USA). The qPCR cycling conditions were: 95°C 30 s, 1 cycle; 95°C 5 s, 55°C 15 s, 72°C 10 s, 40 cycles. The thermal cycler used SimpliAmp A24811 (Applied Biosystems, USA). The qPCR reaction was performed using the HotStar Taq DNA polymerase mix (Transgen Biotech, China). The actin gene was used as a reference gene to ensure the quality of RNA extraction. Homogeneity of the resultant PCR products was confirmed by a melting curve analysis. Graphpad Prism 8.0 software (Graphpad, USA) was used to show the relative expression level of genes. Three replicates were performed for each reaction to account for intra-experiment variation.

## Results

### Transcriptome Data Assessment and Gene Function Annotation

A total of 1,082,140,178 raw reads were obtained. The clean bases after transformation from the sequenced data were 158.01 G. The average clean bases were 8.77 G, and the average sequencing error rate was 0.03%. The GC content ranged from 52.69 and 57.50%, and all Q20 data had greater than 95.01% (Supplementary Table S2). The maximum number of unigenes had an annotated length of 300–500 bp (36,202 unigenes) and 500–1,000 bp (27,433 unigenes) (Supplementary Figure S1). Transcriptome raw data were deposited to the NCBI SRA under accession SRR15676004-15676021 (Bioproject: PRJNA759106). The functional annotation results of seven databases showed that 87,233 genes were annotated, of which 27,156 (31.13%) genes were annotated in the GO database; 26,733 (30.64%) in the NR database; and 17,710 (20.30%) in the SWSS database (Supplementary Figure S2). The genes annotated of GO functional annotation were divided into three major functional categories: Biological process (26 categories), Cellular component (20 categories) and Molecular Function (10 categories) (Supplementary Figure S3). KEGG functional enrichment can reflect the participation of the body in various metabolic pathways. The 11,239 annotated genes included five major categories: Cellular Processes, Environmental Information Processing, Genetic Information Processing, Metabolism, and Organismal Systems. The Signal Transduction, Transport and Catabolism, Translation, and Endocrine System categories genes were more abundant in the ovary of *H. longicornis* (Supplementary Figure S4). The functional enrichment of KOG was divided into 26 categories and included 9,713 genes that were annotated. Of these genes, over 1,000 genes were involved in three categories: General Function Prediction Only, Signal Transduction Mechanisms, and Posttranslational Modification, Protein Turnover, Chaperones (Supplementary Figure S5).

### Transcriptome Differentially Expressed Genes

**Figure 1:**
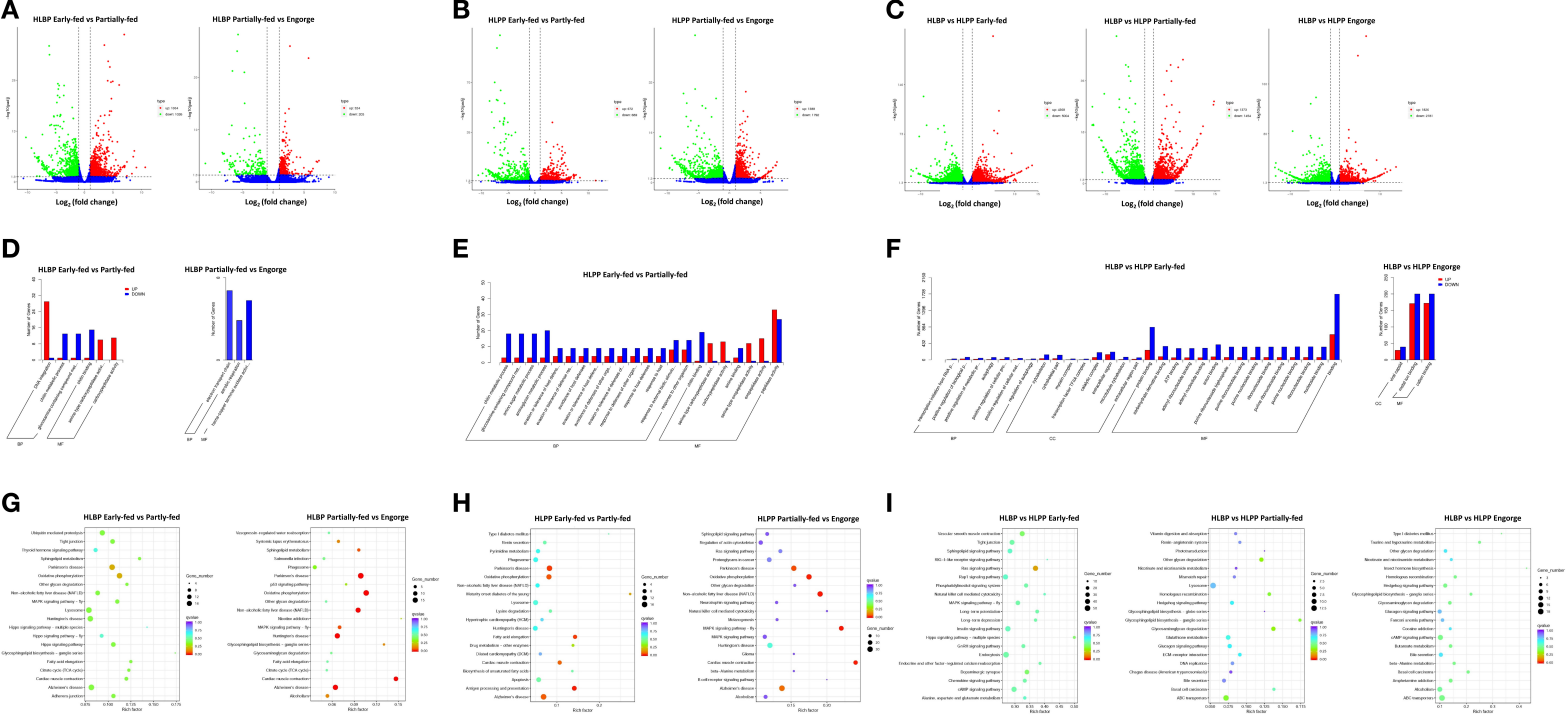
Functional clusterization of differentially expressed genes (DGEs) in the ovary of two reproductive groups of *H. longicornis*. The volcano plots show the fold change in gene expression by the x-axis (log_2_) and the statistical significance of differences by the y-axis (-log_10_). Up regulated and down regulated genes spots are highlighted in green and red circles, respectively. **(A)** Comparison of the *H. longicornis* bisexual population (HLBP) in different feeding stages. **(B)** Comparison of HLPP in different feeding stages. **(C)** Comparison of different reproductive groups. The Gene Ontology (GO) annotation enrichment of differentially expressed genes (DEGs). The histogram shows the up-regulated and down-regulated different categories in red and blue, respectively. **(D)** Comparison of bisexual *H. longicornis* (HLBP) in different feeding stages. **(E)** Comparison of parthenogenetic *H. longicornis* (HLPP) in different feeding stages. **(F)** Comparison of the different reproductive groups. The Kyoto Encyclopedia of Genes and Genomes (KEGG) pathway analysis of differentially expressed genes (DEGs). The colors indicate saliency and size indicates the number of genes in the pathway. **(G)** Comparison of bisexual *H. longicornis* (HLBP) in different feeding stages. **(H)** Comparison of parthenogenetic *H. longicornis* (HLPP) in different feeding stages. **(I)** Comparison of the different reproductive groups.

In the HLBP, the numbers of DEGs in the ovaries of the early-fed to partially-fed *H. longicornis* included 2,030 (1,004 up-regulated and 1,026 down-regulated) genes, and the partially-fed to engorged stage included 527 genes (324 up-regulated and 203 down-regulated) (Figure 1A). In the HLPP, the numbers of DEGs in the ovaries of the *H. longicornis* included 1,361 genes (672 up-regulated and 689 down-regulated) in the early-fed to partially-fed stages, and 3,180 genes (1,388 up-regulated and 1,792 down-regulated) in the partially-fed to engorged stage (Figure 1B). The comparison of different feeding stages between the two reproductive groups showed a large number of DEGs. There were 9,372 (4,368 up-regulated and 5,004 down-regulated) DEGs in the early-fed stage. The number of DEGs in the partially-fed and engorged stages was >1,000, and there were significant differences in transcription regulation between the two reproductive groups during ovarian development (Figure 1C).

### Enrichment of Differentially Expressed Genes

The GO enrichment analysis revealed ten categories of significantly up-regulated and down-regulated genes from the early-fed to partially-fed stages in HLBP. Among them, DNA Integration (29 genes), Serine-type Carboxypeptidase Activity (19 genes) and Serine Hydrolase Activity (19 genes) contained the up-regulated genes, while a small number of Chitin Binding (15 genes), Chitin Metabolic Process (13 genes) and Glucosamine-containing Compound Metabolic (13 genes) related genes were down-regulated. There was no category of significantly up-regulated genes in the partially-fed to engorged stages of the HLBP (Figure 1D). In the HLPP, there were many up-regulated genes in the early-fed to partially-fed status. These genes were in categories such as Exopeptidase Activity (15 genes), Carboxypeptidase Activity (13 genes) and Serine-type Carboxypeptidase Activity (12 genes). The functional categories of Aminoglycan Metabolic (20 genes), Chitin Metabolic (19 genes), Amino Sugar Metabolic (18 genes) and Glucosamine Containing (18 genes) were mainly down-regulated (Figure 1E). There was no category of DEGs in the partially-fed to engorged stages of the HLPP. The comparison of the two reproductive groups indicated that the early-fed stage had more GO categories of DEGs. The early-fed stage was involved in more extensive functions, while the Binding (2,389 genes) and Protein Binding (1,116 genes) were significantly enriched. The comparison of engorged stage DEGs involved three categories: Cation Binding (372 genes), Metal Ion Binding (371 genes) and Viral Capsid (68 genes). The enrichment of DEGs in the partially-fed stage was not found functional category, suggest that the gene transcript patterns were more similar in this stage (Figure 1F).

The KEGG pathway analysis of HLBP showed that a large number of ovarian DEGs involved Lysosome (19 genes), Oxidative Phosphorylation (18 genes) and Ubiquitin Mediated Proteolysis (16 genes) in the early-fed to partially-fed stages, and the DEGs of the partially-fed to engorged stages mainly involved Oxidative Phosphorylation (17 genes), MAPK Signaling Pathway-fly (7 genes) and Sphingolipid Metabolism (5 genes) (Figure 1G). The DEGs of HLPP that involved Oxidative Phosphorylation (13 genes), Lysosome (12 genes), Antigen Processing and Presentation (11 genes) and Apoptosis (11 genes) were more abundant in the early-fed stage in the ovary tissue, and the partially-fed to engorged stages had a higher number DEGs in Oxidative Phosphorylation (28 genes) and MAPK Signaling Pathway-fly (22 genes) (Figure 1H). The DEGs in the comparison between HLBP and HLPP were mainly related to the Endocytosis (50 genes), Insulin Signaling Pathway (42 genes), Ras Signaling Pathway (41 genes), cAMP Signaling Pathway (41 genes), ABC Transporters (18 genes) and Lysosome (13 genes) in different stages, but the significance of the DEGs had lower Q-values (Figure 1I).

### Function Annotation of Proteome Data

The ovarian samples from two groups of *H. longicornis* at different feeding stages were quantitatively assessed by mass spectrometry. Proteome raw data were deposited to the iProX under accession PXD029485. A total of 1,902–2,113 proteins were annotated from the ovary transcriptome database of *H. longicornis* (Supplementary Table S2). Correlation analysis showed that HLBP was higher than 0.9 and HLPP was higher than 0.8, indicating that the sample protein expression has good repeatability (Supplementary Figure S6). The proteome data were then annotated in GO, KEGG and KOG. GO annotation found that many genes, including Binding, Cellular Process, Metabolic Process, Catalytic Activity, and Single-organism Process, were related to ovarian development (Supplementary Figure S7). KEGG enrichment analysis showed that Translation, Folding, Sorting and Degradation, Signal Transduction, Transport and Catabolism had many genes involved in ovary development (Supplementary Figure S8). The classifications of Posttranslational Modification, Protein Turnover, Chaperones, General Function Prediction Only and Signal Transduction Mechanisms accounted for the highest proportion of KOG annotations (Supplementary Figure S9).

### Proteomics Analysis of Differentially Expressed Proteins

**Figure 2:**
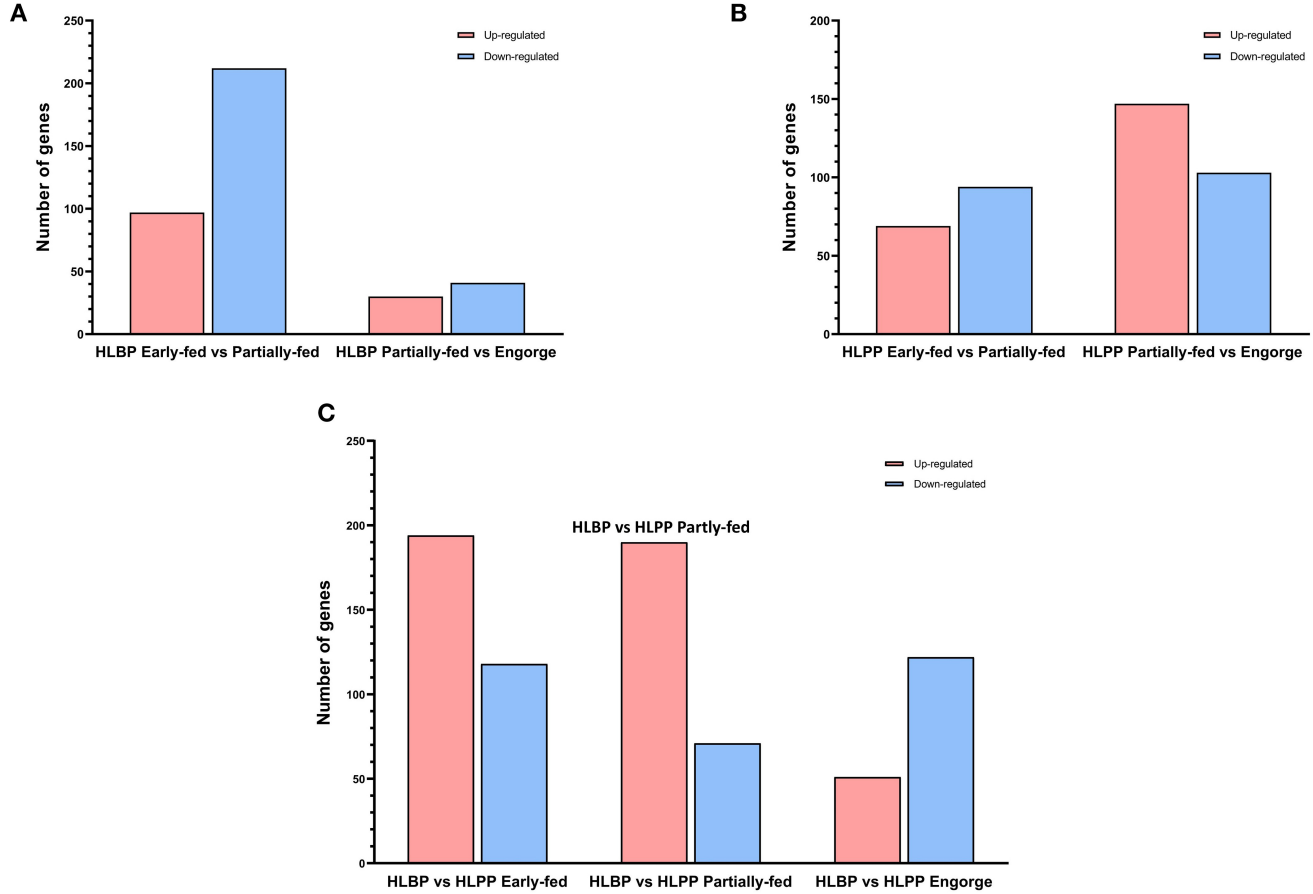
Differentially expressed proteins (DEPs) of *H. longicornis* ovary. The histogram shows the up-regulated and down-regulated different categories in red and blue, respectively. **(A)** Comparison of bisexual *H. longicornis* (HLBP) in different feeding stages. **(B)** Comparison of parthenogenetic *H. longicornis* (HLPP) in different feeding stages. **(C)** Comparison of the different reproductive groups.

DEPs in the early-fed to partially-fed stages of HLBP included 309 (97 up-regulated and 212 down-regulated) proteins, and the partially-fed to engorged stages included 71 proteins (30 up-regulated and 41 down-regulated). In the HLPP, DEPs included 163 proteins (69 up-regulated and 94 down-regulated) in the early-fed to partially-fed stages, and 250 proteins (147 up-regulated and 103 down-regulated) in the partially-fed to engorged stages. Comparison of the two reproductive groups showed that the DEPs were more abundant in the partially-fed stage (194 up-regulated and 118 down-regulated) and engorged stage (190 up-regulated and 71 down-regulated), both of which were higher than in the early-fed stage (51 up-regulated and 122 down-regulated) (Figure 2).

### Enrichment of Differentially Expressed Proteins

**Figure 3:**
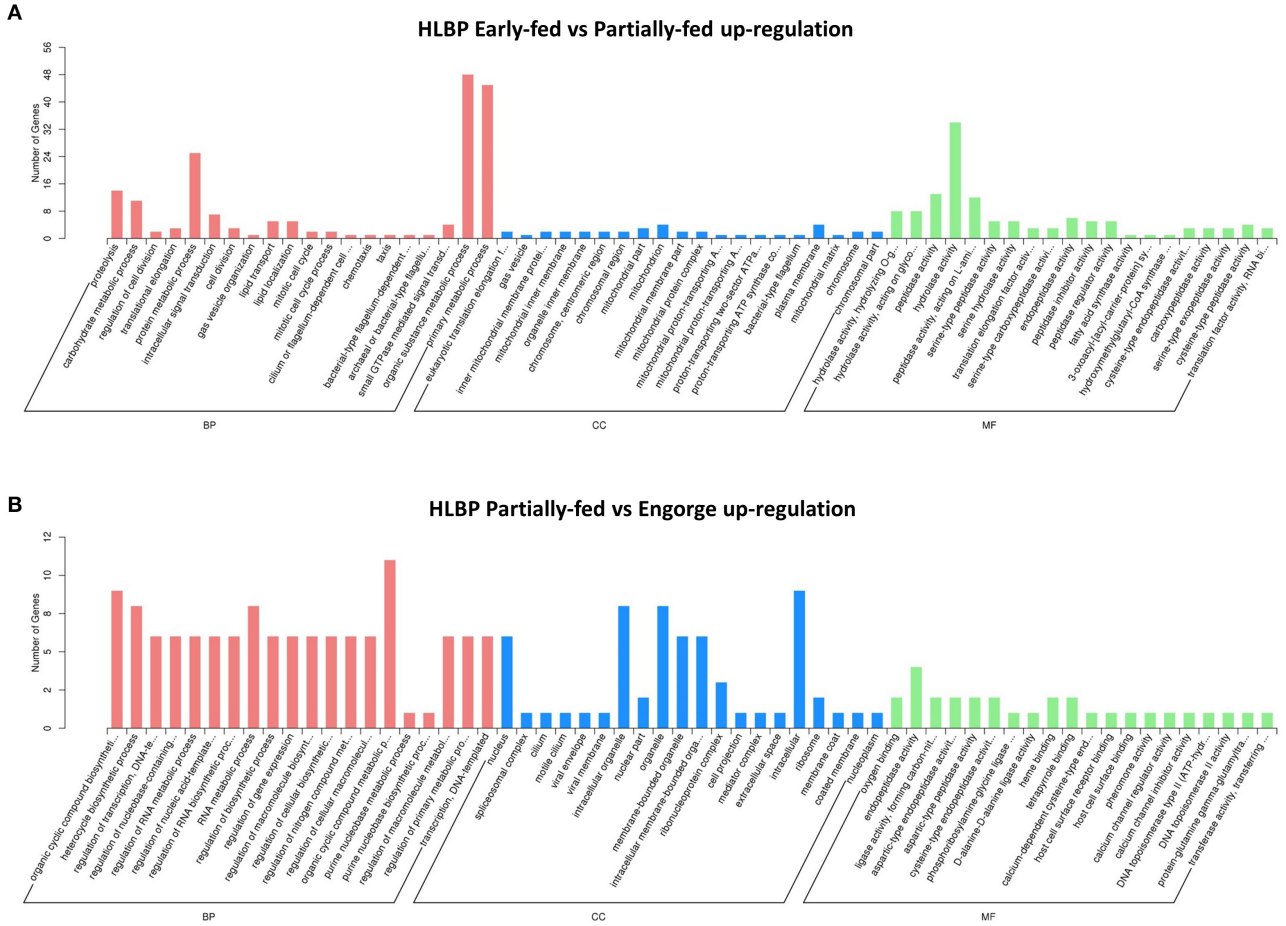
Gene Ontology (GO) annotation enrichment of upregulated proteins in bisexual *H. longicornis* (HLBP) at different feeding stages. The x-axis shows the functional categories of the proteins, and the y-axis shows the number of increased proteins. **(A**) Comparison of bisexual *H. longicornis* (HLBP) in early-fed to partially-fed stages. **(B)** Comparison of bisexual *H. longicornis* (HLBP) in partially-fed to engorge stages.

**Figure 4:**
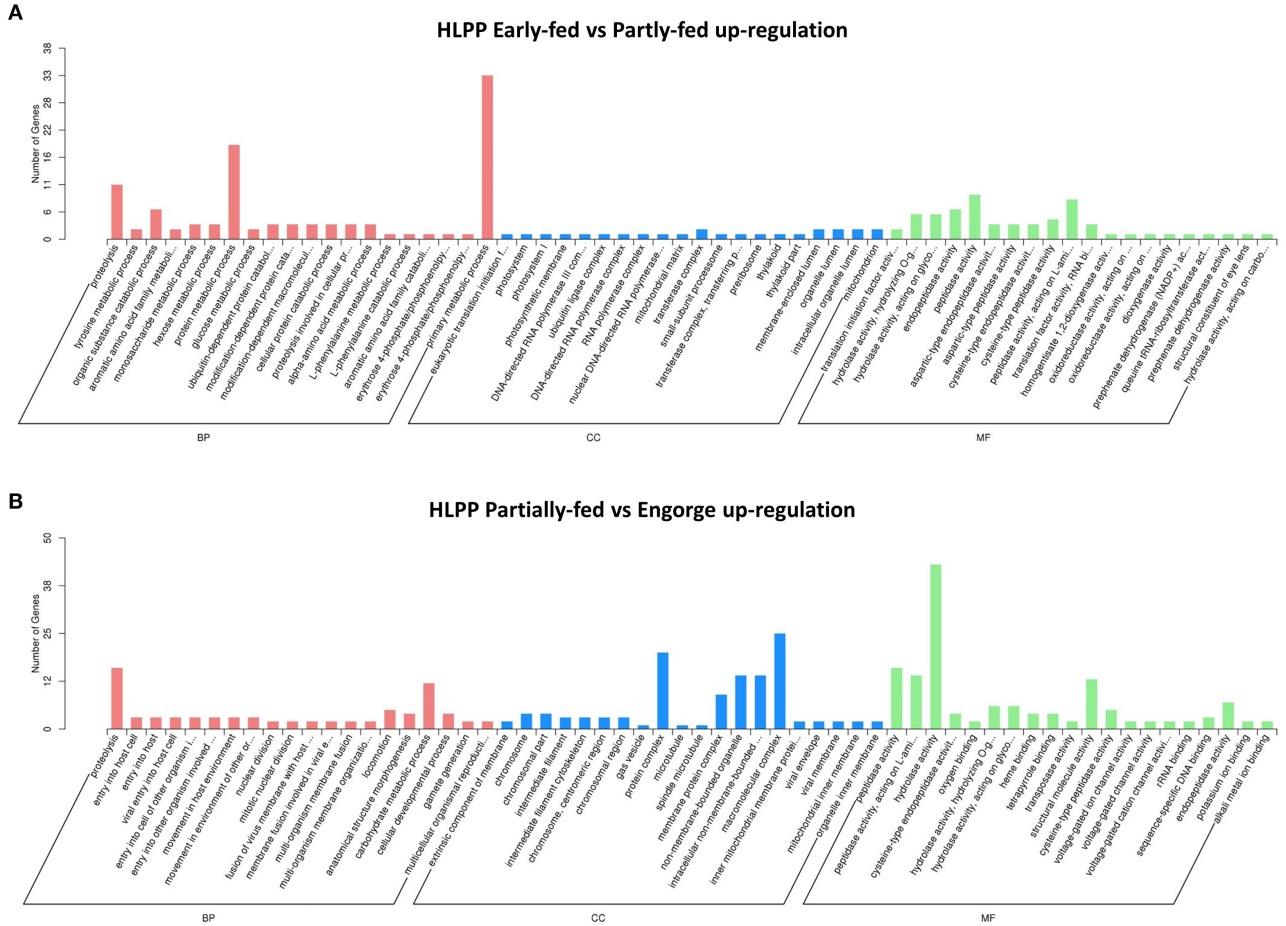
Gene Ontology (GO) annotation enrichment of upregulation proteins in parthenogenetic *H. longicornis* (HLPP) at different feeding stages. The x-axis shows the functional categories of the proteins, and the y-axis shows the number of increased proteins. **(A**) Comparison of parthenogenetic *H. longicornis* (HLBP) in early-fed to partially-fed stages. **(B)** Comparison of parthenogenetic *H. longicornis* (HLBP) in partially-fed to engorge stages.

The functional categories of proteome were similar to those in transcriptome annotation. The number of DEPs was significantly less than that of the transcriptome. The proteins were analyzed using tertiary functional annotation in the GO database, and results showed the Organic Substance Metabolic Process (48 proteins), Primary Metabolic Process (45 proteins), Protein Metabolic Process (25 proteins) and Hydrolase Activity (34 proteins) in the early-fed to partially-fed stages, as well as Organic Cyclic Compound Metabolic Process (nine proteins), were involved in up-regulation during the partially-fed to engorged stages (Figure 3). In the HLPP, more proteins related to Primary Metabolic Process (33 proteins), Protein Metabolic Process (19 proteins) and Proteolysis (11 proteins) were up-regulated in the early-fed to partially-fed stages. Hydrolase Activity (43 proteins), Macromolecular Complex (25 proteins) and Protein Complex (20 proteins) were associated with a greater number of proteins from the partially-fed to engorged stages (Figure 4).

### Quantitative Verification of Expressions

**Figure 5:**
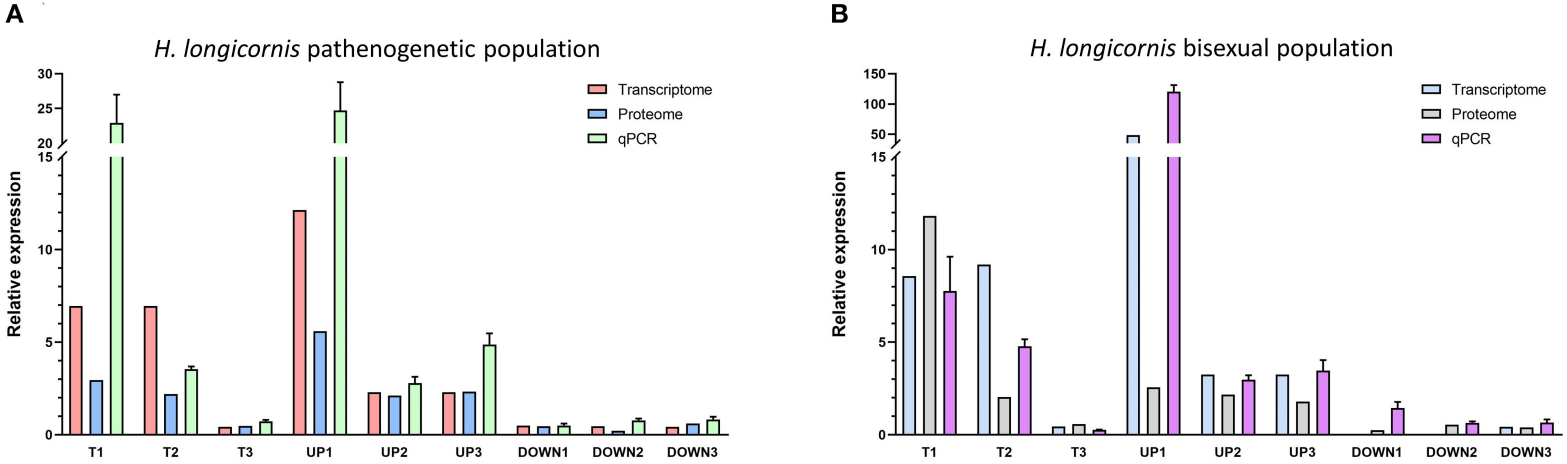
Quantitative PCR of differentially expressed genes and proteins in the different feeding stages of bisexual and parthenogenetic groups. Data from 17 of the 18 groups followed the same expression trend as the transcriptome and proteome data. **(A)** Quantitative analysis of bisexual *H. longicornis* (HLBP) for Differentially expressed proteins (DEPs). **(B)** Quantitative analysis of parthenogenetic *H. longicornis* (HLBP) for Differentially expressed proteins (DEPs).

The analysis of transcriptome and proteome data was integrated to explore overlap proteins with similar expression. A total of 11 proteins (seven up-regulated proteins and four down-regulated) were screened in the HLBP, and 25 proteins (21 up-regulated proteins and four down-regulated) in the HLPP (Table 1). Three proteins with the same expression were found in the two reproductive groups. The functional annotations were: transferrin receptor (T1), cathepsin L (T2), and WW domain binding protein (T3). The quantitative analysis showed that 17 of 18 groups followed the same expression trend as the transcriptome and proteome data, indicating that the protein expression trend obtained with the screening both transcriptome and proteome data had high accuracy (Figure 5).

**Table 1:** Differential expression genes and proteins with the same expression trends in the ovary of the HLBP and HLPP.

**Transcript**	**Putative function**	**Genbank ID**
**HLB up**		
Cluster-24273.0	Conserved hypothetical protein [*Ixodes scapularis*]	EEC14909.1
Cluster-16082.0	Transferrin receptor, putative [*Ixodes scapularis*]	XP_002407670.1
Cluster-19607.16368	Alpha-L-fucosidase, putative [*Ixodes scapularis*]	EEC16229.1
Cluster-19607.18741	Conserved hypothetical protein [*Ixodes scapularis*]	XP_002412983.1
Cluster-19607.33672	Cathepsin L [*Rhipicephalus haemaphysaloides*]	ALQ43545.1
Cluster-19607.44128	Secreted protein, putative [*Ixodes scapularis*]	EEC17184.1
Cluster-19607.47249	Beta-N-acetylhexosaminidase, putative [*Ixodes scapularis*]	XP_002400043.1
**HLB down**		
Cluster-19607.24316	Oligopeptidase, putative [*Ixodes scapularis*]	XP_002405238.1
Cluster-19607.31271	Succinyl-CoA synthetase, beta subunit, putative [*Ixodes scapularis*]	XP_002411799.1
Cluster-19607.34970	KH domain RNA binding protein, putative [*Ixodes scapularis*]	XP_002435182.1
Cluster-19607.38572	WW domain-binding protein, putative [*Ixodes scapularis*]	EEC06693.1
**HLP up**		
Cluster-19607.33672	Cathepsin L [*Rhipicephalus haemaphysaloides*]	ALQ43545.1
Cluster-19607.22090	Lysozyme [*Haemaphysalis longicornis*]	BAK20441.1
Cluster-10378.0	31 kDa Antigen [*Plasmodium berghei*]	AAO22146.1
Cluster-16082.0	Transferrin receptor, putative [*Ixodes scapularis*]	XP_002407670.1
Cluster-19607.21762	Vitellogenin [*Dermacentor variabilis*]	AAW78557.2
Cluster-19607.43922	Hemolectin, putative, partial [*Ixodes scapularis*]	EEC02351.1
Cluster-19607.25799	Flavonol reductase / cinnamoyl-CoA reductase, putative, partial [*Ixodes scapularis*]	EEC14439.1
Cluster-19607.41664	Unconventional myosin-Id-like [*Centruroides sculpturatus*]	XP_023227103.1
Cluster-19607.26478	Heat Shock Protein 20-like protein [*Euroglyphus maynei*]	OTF77562.1
Cluster-19607.33273	Protein required for fusion of vesicles in vesicular transport, alpha-SNAP, putative [*Ixodes scapularis*]	XP_002416615.1
Cluster-19607.29323	Ras-related protein Rac1 [*Limulus polyphemus*]	XP_013779934.1
Cluster-19607.24098	TPA_inf: hypothetical secreted protein 790 [*Amblyomma variegatum*]	DAA34730.1
Cluster-19607.33376	Glutathione S-transferase [*Haemaphysalis longicornis*]	AAQ74441.1
Cluster-19607.37239	Myosin heavy chain, muscle isoform X10 [*Parasteatoda tepidariorum*]	XP_015930663.1
Cluster-19607.31582	Muscle-specific protein 20 [*Parasteatoda tepidariorum*]	XP_015905796.1
Cluster-19607.47213	Vacuolar ATP synthase subunit E, putative [*Ixodes scapularis*]	XP_002405182.1
Cluster-19607.20809	Phosphatidate phosphatase LPIN2-like [*Limulus polyphemus*]	XP_013782870.1
Cluster-19607.39621	Anaphase-promoting complex subunit 2-like [*Limulus polyphemus*]	XP_013794784.2
Cluster-19607.36816	Putative heat shock-related protein [*Dermacentor variabilis*]	AAO92281.1
Cluster-19607.35390	Ras-related protein Rab-10-like [*Centruroides sculpturatus*]	XP_023234078.1
Cluster-19607.19998	Uncharacterized protein LOC108914600 [*Anoplophora glabripennis*]	XP_023312689.1
**HLP down**		
Cluster-22462.0	Ferritin 2 [*Haemaphysalis longicornis*]	BAN13552.1
Cluster-19607.38572	WW domain-binding protein, putative [*Ixodes scapularis*]	EEC06693.1
Cluster-19607.48479	Aspartic protease [*Haemaphysalis longicornis*]	ABV71391.1
Cluster-19607.34957	Methylmalonate-semialdehyde dehydrogenase (acylating), mitochondrial, partial [*Stegodyphus mimosarum*]	KFM56888.1

## Discussion

We explored the molecular mechanisms of ovarian development in *H. longicornis* during different feeding stages using the transcriptome and proteome. Further functional annotation of the DEGs led to the identification of potential functional genes associated with ovarian development in ticks. GO enrichment showed that many genes are related to signal transduction, cell differentiation and catalytic activity. The type of gene expression suggests rapid ovarian development of *H. longicornis* during feeding. KEGG pathway analysis showed that catabolic processes, signal transduction, translation modification and lipid metabolism may play important roles in *H. longicornis* ovary nutrient digestion and ovary anabolism. KOG analysis showed that there were many genes related to signal transduction mechanisms and protein posttranslational modification. This indicates that many genes in *H. longicornis* were involved in transcriptional activation, cell differentiation and cell migration.

The ovarian development of HLBP during different feeding stages triggered many up-regulated genes in various GO categories. DNA fusion genes may play a role in ovarian cell proliferation and oocyte meiosis. Lysosomes participate in the digestion of blood nutrients through a series of hydrolases and phospholipases (32, 33). Carboxypeptidase related genes are involved in protein degradation and synthesis, and metabolites provide nutrition for ovarian and embryonic development (23). Oxidative Phosphorylation genes are involved in the energy release of nutrients, and their increased activity supports the energy metabolism of ovarian development (34). Ubiquitin mediated proteolysis is involved in gene expression and regulation of ovarian development at the chromatin structural level (35), as well as in the early degradation of many proteins. The MAPK Signaling Pathway is regulated by juvenile hormones (36), which are involved in the structural reorganization of ovarian germ cells during meiosis (37), affecting spindle assembly and mitosis stability (38). Genes in sphingolipid metabolism are important in meiosis and maturation of germ cells (39), and may be involved in cell proliferation, membrane synthesis and antioxidation in tick ovary development (40). Genes related to the ovarian development of HLPP during feeding were up-regulated in the Oxidative Phosphorylation and MAPK Signaling Pathway, and the related genes may play a conservative role in the *H. longicornis* ovary. Further, HLPP expresses many up-regulated genes in peptidase activity related pathways, and peptidase is a proteolytic enzyme important in insect growth and development (41, 42). Serine protease is a major immune-related protein in insects; it is involved in the regulation of blood coagulation, the activation of cytokines and the induction of oocyte maturation (43, 44).

The number of DEGs in the HLBP ovary during the feeding stages was greater than that in the HLPP, which indicated that there were significant differences in the transcription patterns of ovarian development between the different reproductive strategies. The greatest difference in transcription patterns between the two reproductive groups was found at the early feeding status. The results of GO enrichment showed that there were many Binding protein pathways in the early-feeding status, suggesting that there were differences in the transcriptional mechanism. Among the DEGs in KEGG pathway analysis, many were found in PI3K-Akt Signaling Pathway. The PI3K protein family is involved in the regulation of cell proliferation, differentiation, apoptosis and other cell functions, which may promote the proliferation and development of oocytes (34). The cAMP Signaling Pathway is regulated by hormones, which can activate adenylate cyclase to synthesize ATP. This is an important conserved pathway of organism energy metabolism. ABC Transporter is a transmembrane protein, which can use ATP hydrolysis to complete transmembrane transport (45). Additionally, Zinol and Hormone Regulation pathways can regulate the development process of the ovary. The activity of related genes plays a key role in oogenesis and oocyte maturation (46). The DEGs in feeding stages between the HLBP and HLPP mean that significant changes have taken place in the ovarian tissue transcription mode and gene function.

GO enrichment of the HLBP and HLPP ovarian proteome showed that there were similarities in the functional types between genes and proteins. However, KOG analysis showed that there were differences between the proteome and transcriptome. The lower number of General Function prediction proteins was only found in the proteome, whereas the proportion of proteins with Posttranslational Modification, Protein Turnover, Chaperones, Translation, Ribosomal Structure and Biogenesis Related Functions was increased. KEGG pathway analysis showed that there were many functional genes in Transcription Folding, Sorting and Degeneration Proteins. In the HLBP, a relatively high number of DEPs was found from the early-fed to partially-fed status, but in the HLPP, a greater number of DEPs was found from the partially-fed to engorgement stages. At the beginning of feeding, a rapid increase of protein expression was found in the HLPP, which was not observed in the HLBP. This could result from unmated females of HLBP that could not reach engorgement and complete ovarian development due to the lack of mating or the absence of stimulation by male proteins. This physiological change probably results in a significantly different protein expression patterns during the feeding stages between the two reproductive groups of *H. longicornis*, and both the proteome and transcriptome results coincided with this feature. Proteome comparison between the two reproductive groups showed that the number of DEPs was the lowest in the early-fed stage; however, the number of DEGs was the highest in the transcriptome. The differing results of the transcriptome and proteome may be related to post-transcriptional modification or translation regulation, which indicates that there may be no definite relationship between genes in organisms at the transcriptional and translation levels (47). The expression trends of the transcriptome and proteome were verified through quantitative PCR, and the related genes could be actively involved in important physiological processes, such as nutrition metabolism, cell proliferation and immune response.

**Figure 6:**
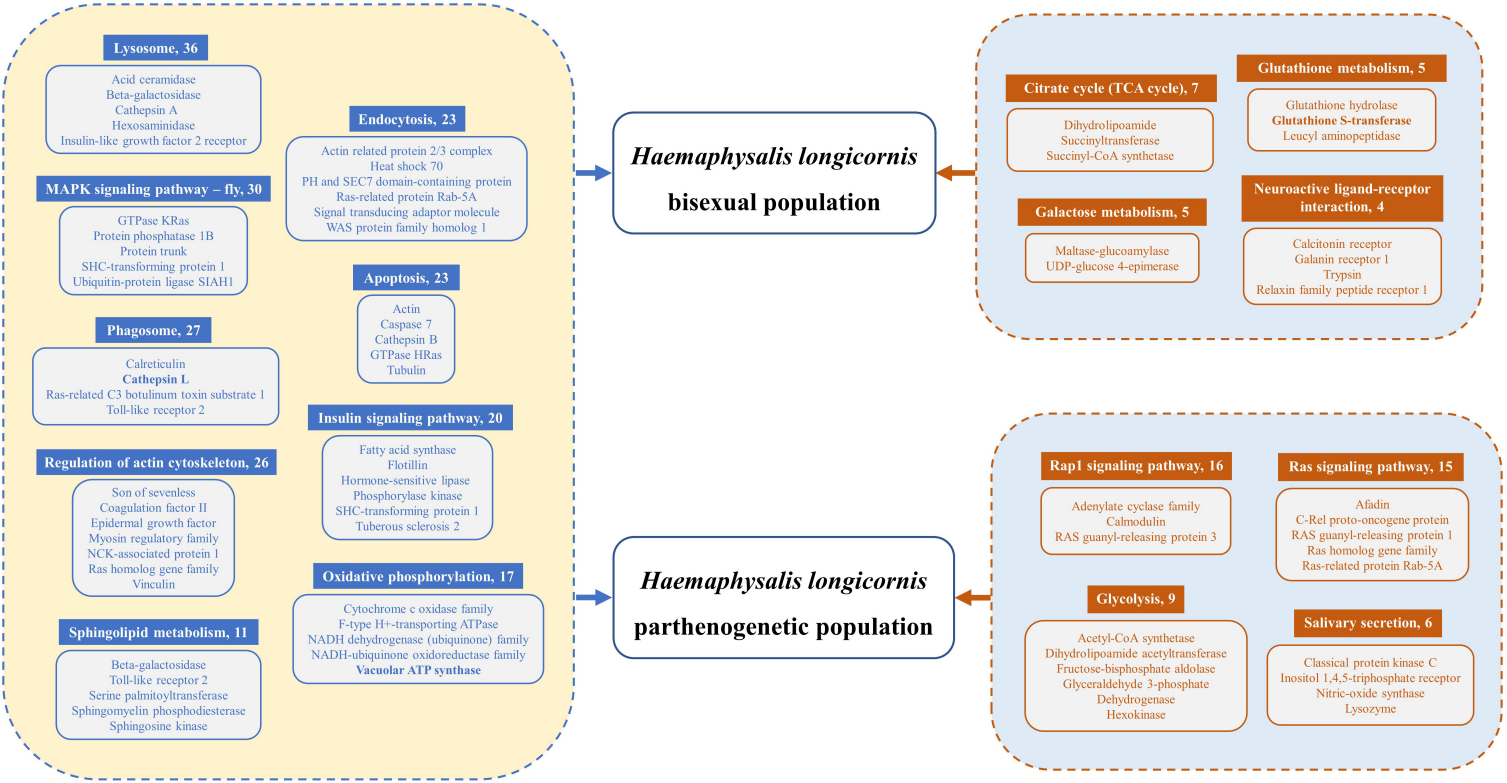
Differentially expressed genes (DEGs) of functional pathways of HLBP and HLPP. The common upregulated genes of HLBP and HLPP are on the left side of the legend. The exclusive upregulated genes of HLBP or HLPP are on the right side of the legend. Numbers following the pathway names indicate the quantity of transcripts.

Functional annotation pathway analysis showed that the common upregulated genes of two reproductive groups were involved in Lysosome, MAPK Signaling Pathway, Phagosome, Regulation of Actin Cytoskeleton, Endocytosis, Apoptosis, Insulin Signaling Pathway, Oxidative Phosphorylation, and Sphingolipid Metabolism (Figure 6). Our findings suggest that, in addition to the increase in cell metabolism, the activities of genes related to lipid metabolism, hormone regulation and protein synthesis were also enhanced in the ovary of *H. longicornis* during different feeding stages, which provided the physiological basis for ovarian development (48). Furthermore, some genes were up-regulated specifically in each group. The upregulated genes of HLBP were mainly included in Citrate cycle (TCA cycle), Galactose metabolism, Glutathione metabolism, and the Neuroactive ligand-receptor interaction pathway (Figure 6). The HLBP showed accelerated sugar metabolism for ovarian development, which indicated an enhanced consumption rate (49). In the HLPP, the main upregulated genes were annotated as Rap1 signaling pathway, Ras signaling pathway, Glycolysis, and Salivary secretion (Figure 6). The genes involved in RAS signaling lead to constantly activated cell proliferation and differentiation (50).

Seven proteins were up-regulated simultaneously by the ovarian transcriptome and proteome from the early-fed to partially-fed stages of HLBP. Transcription factors were up-regulated in the early-fed to partially-fed stages of the *H. longicornis*, which may be affected by the transcription activity of hydrolases. Transferrin receptors mediate transferrin from outside into the cell and participate in signal transduction and early oocyte material transport (51). During ovarian development, cathepsin L is involved in transcriptional regulation of oocyte maturation (52) and plays an important role in nutrition digestion and immunity of the insect ovary (41, 42). Secreted protein may be important in tick hematopoietic attachment and antigen recognition (53–55) and participate in follicular cell development. The up-regulated expression of two glycosidases can increase carbohydrate metabolism activity (Alpha-L-fucosidase and Beta-N-acetylhexo–saminidase), which may be involved in the digestion and transport of blood nutrients (56).

The two up-regulated proteins (transferrin receptor and cathepsin L) of HLBP in the early-fed status were also present in HLPP, suggesting that these proteins play a conserved role in ovarian development of the two reproductive groups. There were more up-regulated proteins from the early-fed to partially-fed stages of HLPP. Lysozyme is an antimicrobial peptide involved in blood digestion and immune responses (57); it may affect the development of eggs (58). Vitellogenin is the precursor of vitellin, which is the main nutrient of fertilized eggs (59). Heat shock protein is the most important protein family in insect antagonistic stress response, and it can promote the repair of damaged cells (60). The expression of heat shock protein is increased in ovaries and oocytes in genital development (61). We found two heat shock family proteins (heat shock protein 20-like protein and heat shock-related protein) in the HLPP, which may be involved in oxidative stress protection and yolk protein synthesis during ovarian development of *H. longicornis* (62). The glutathione S-transferase upregulation in HLPP may indicate an increase in ovarian antioxidant activity; it is an important enzyme in the process of nutrient absorption and digestion (63) and plays a protective role in the rapid development of ovarian tissue. During oocyte development, myosin participates in the transport of cell nutrients while germ cells with myosin deficiency may have serious defects in cell proliferation and cytoplasmic division (61, 64, 65). Vacuolar ATP synthase subunit E may be involved in the metabolism of energy substances in *H. longicornis* ovary cells. The increased expression of ATP synthetase may play an important role in ovarian development and cell proliferation (56). Phosphatidate phosphatase LPIN2-like is most likely linked to ovarian lipid transport and ovarian cell development (66) and is a component of lipid transmission during ovarian development (34). The differential expression profile of DEGs and DEPs between HLPP and HLBP suggest complex underlying gene expression regulation mechanisms, and the greater number of DEGs and DEPs observed in HLPP may facilitate faster initiation of egg laying and individual development. Some genes that were found important for insect ovarian development, such as Vitellogenin and Lysozyme, were not detected simultaneously in HLBP and HLPP during early ovarian development.

The ovarian transcription patterns of two reproductive groups of *H. longicornis* at different feeding stages were different. Differences in gene expression have a profound influence on phenotypic trait. During female insect reproduction, various hormone types are closely related to vitellogenesis, oocyte maturation and oocyte maturation. Different insects have different reproductive strategies and regulatory pathways (48). An attempt was made in this study to screen for some sex-determining genes. However, the genetic differentiation signals of parthenogenesis in ticks are difficult to identify. Among the differentially expressed genes in the ovary of the different reproductive groups, no sex control genes were identified. Most genes were related to developmental and metabolic pathways. Ticks and TBDs are a medical and veterinary public health challenge, and are usually controlled through the use of repellents and acaricides. Research on vaccination strategies to protect humans, pets, and domestic animals from ticks and tick-borne pathogens has accelerated through high-throughput analyses. Given that tick ovarian development plays an integral role in the reproduction of their populations and the transmission of pathogens, comparative analysis of different blood-sucking stages provides valuable insights for screening key genes for ovarian development. These genes may provide the basis for the development of more effective anti-tick vaccines. In additional, assessing the role of these genes in regulating reproductive development and exploring their complex interactions in reproductive regulation will help reveal the mechanism of tick reproduction regulation and the evolutionary mechanism of parthenogenesis.

## Conclusions

Ovarian development genes and proteins were annotated and analyzed from bisexual and parthenogenetic *H. longicornis* in different feeding stages. The genes were mainly concentrated in oxidizing phosphorylation, signal transduction, cell differentiation, translation modification, substance metabolism and catalytic activity. DEGs of functional pathway analysis indicated Lysosome, MAPK Signaling Pathway, Phagosome, Regulation of Actin Cytoskeleton, Endocytosis, Apoptosis, Insulin Signaling Pathway, Oxidative Phosphorylation and Sphingolipid Metabolism were most abundant in the feeding stages. There were many DEGs in the PI3K-Akt Signaling Pathway, cAMP Signaling Pathway and ABC Transporter, indicating that there were different transcription patterns or gene functions in the ovarian development of the two reproductive groups. A large number of up-regulated genes in the ovaries of HLBP and HLPP, such as cathepsin L, secreted protein, glycosidase, lysozyme, vitellogenin, heat shock protein, glutathione S transferase, myosin and ATP synthase, are worthy of advanced research regarding molecular function. These results may provide insights into the molecular mechanism of tick reproductive system development as well as molecular targets for tick control.

## Data Availability Statement

The datasets presented in this study can be found in online repositories. The names of the repository/repositories and accession number(s) can be found here: BioProject, PRJNA759106; iProX, PXD029485.

## Author Contributions

ZY and JL conceived the idea. HW and TiW carried out the fieldwork. TiW, ToW, MeZ, XS, and MiZ performed the experiments. TiW and XY analyzed the data and drafted the manuscript. All authors contributed to the article and approved the submitted version.

## Funding

This work was supported by the Natural Science Foundation of Hebei province (No. C2021423003), the Doctoral Research Fund of Hebei College of Traditional Chinese Medicine (No. 3010105236), and the Talent Team Construction Project of Hebei College of Traditional Chinese Medicine.

## Conflict of Interest

The authors declare that the research was conducted in the absence of any commercial or financial relationships that could be construed as a potential conflict of interest.

## Publisher's Note

All claims expressed in this article are solely those of the authors and do not necessarily represent those of their affiliated organizations, or those of the publisher, the editors and the reviewers. Any product that may be evaluated in this article, or claim that may be made by its manufacturer, is not guaranteed or endorsed by the publisher.

## Supplementary Material

Supplementary Figure S1 Length distribution of unigenes.

Supplementary Figure S2 Gene annotation results of seven databases.

Supplementary Figure S3 Gene Ontology (GO) annotation of *H. longicornis* ovary genes.

Supplementary Figure S4 The euKaryotic Ortholog Group (KOG) annotation of *H. longicornis* ovary genes.

Supplementary Figure S5 Kyoto Encyclopedia of Genes and Genomes (KEGG) annotation of *H. longicornis* ovary genes.

Supplementary Figure S6 Correlation analysis of mass spectrometry data in ovaries of the two reproductive groups of *H. longicornis*.

Supplementary Figure S7 Gene Ontology (GO) annotation of *H. longicornis* ovary proteins.

Supplementary Figure S8 The euKaryotic Ortholog Group (KOG) annotation of *H. longicornis* ovary proteins.

Supplementary Figure S9 Kyoto Encyclopedia of Genes and Genomes (KEGG) annotation of *H. longicornis* ovary proteins.

Supplementary Table S1 Primer information of quantitative PCR genes.

Supplementary Table S2 Data acquisition and assembly results of the transcriptome data and proteome.
