# Supplementary figures and images for: The Ovarian Development Genes of Bisexual and Parthenogenetic Haemaphysalis longicornis Evaluated by Transcriptomics and Proteomics
Source: Front Vet Sci. 2021 Dec 15;8:783404. doi: 10.3389/fvets.2021.783404 (PMC8714755; doi:10.3389/fvets.2021.783404)

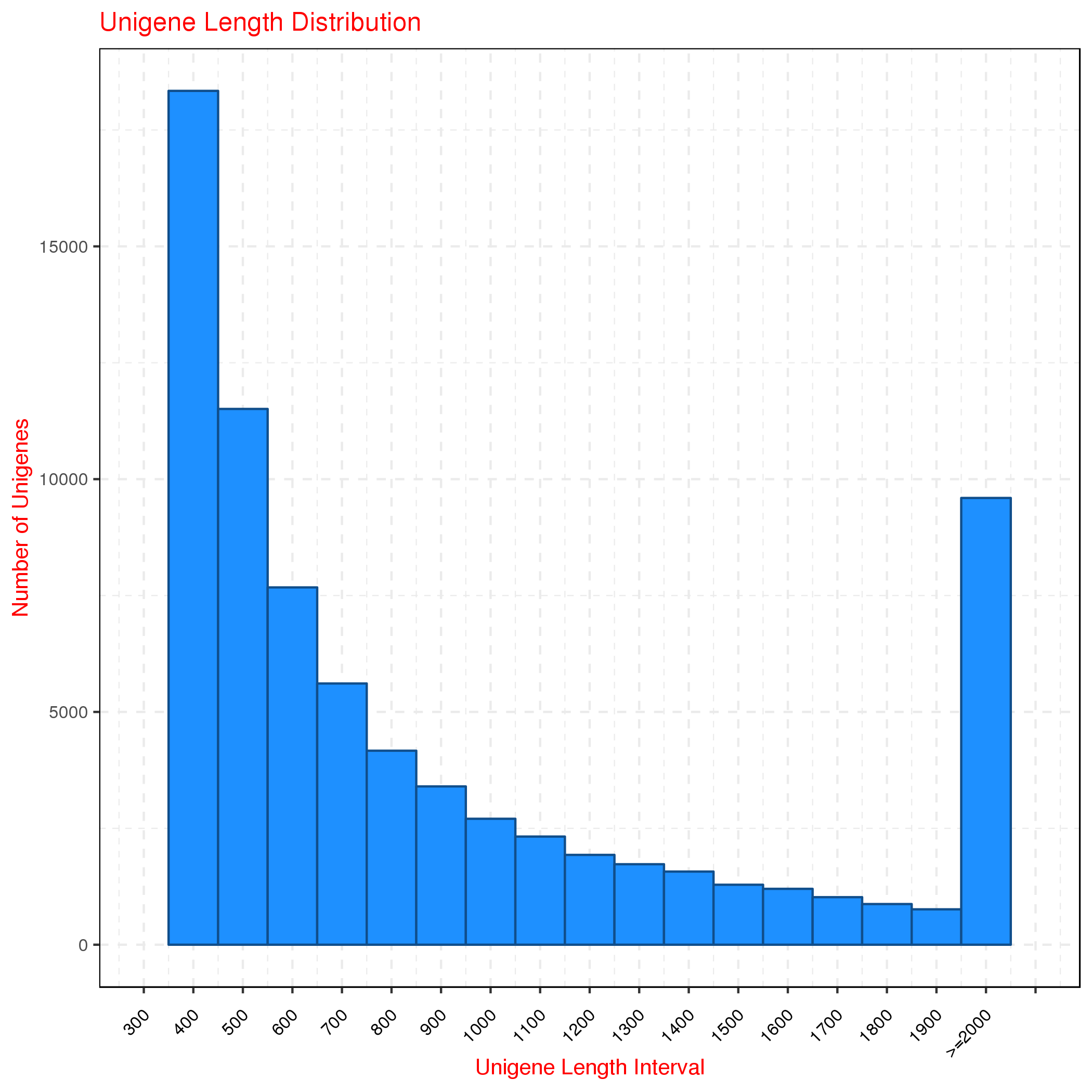

Supplement: Supplementary Figure S1 — Length distribution of unigenes. [file Image_1.PNG]

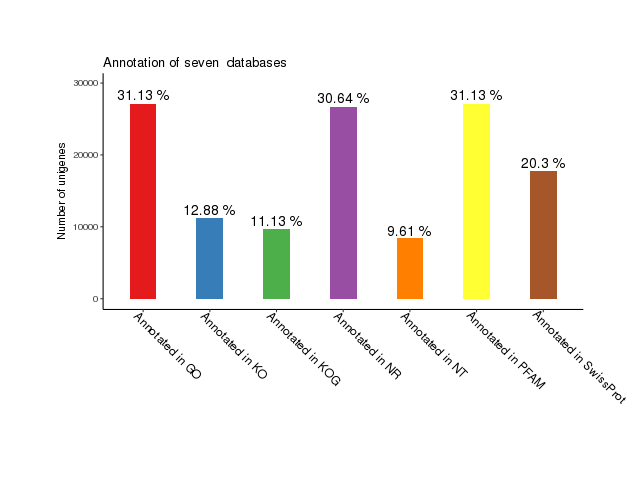

Supplement: Supplementary Figure S2 — Gene annotation results of seven databases. [file Image_2.PNG]

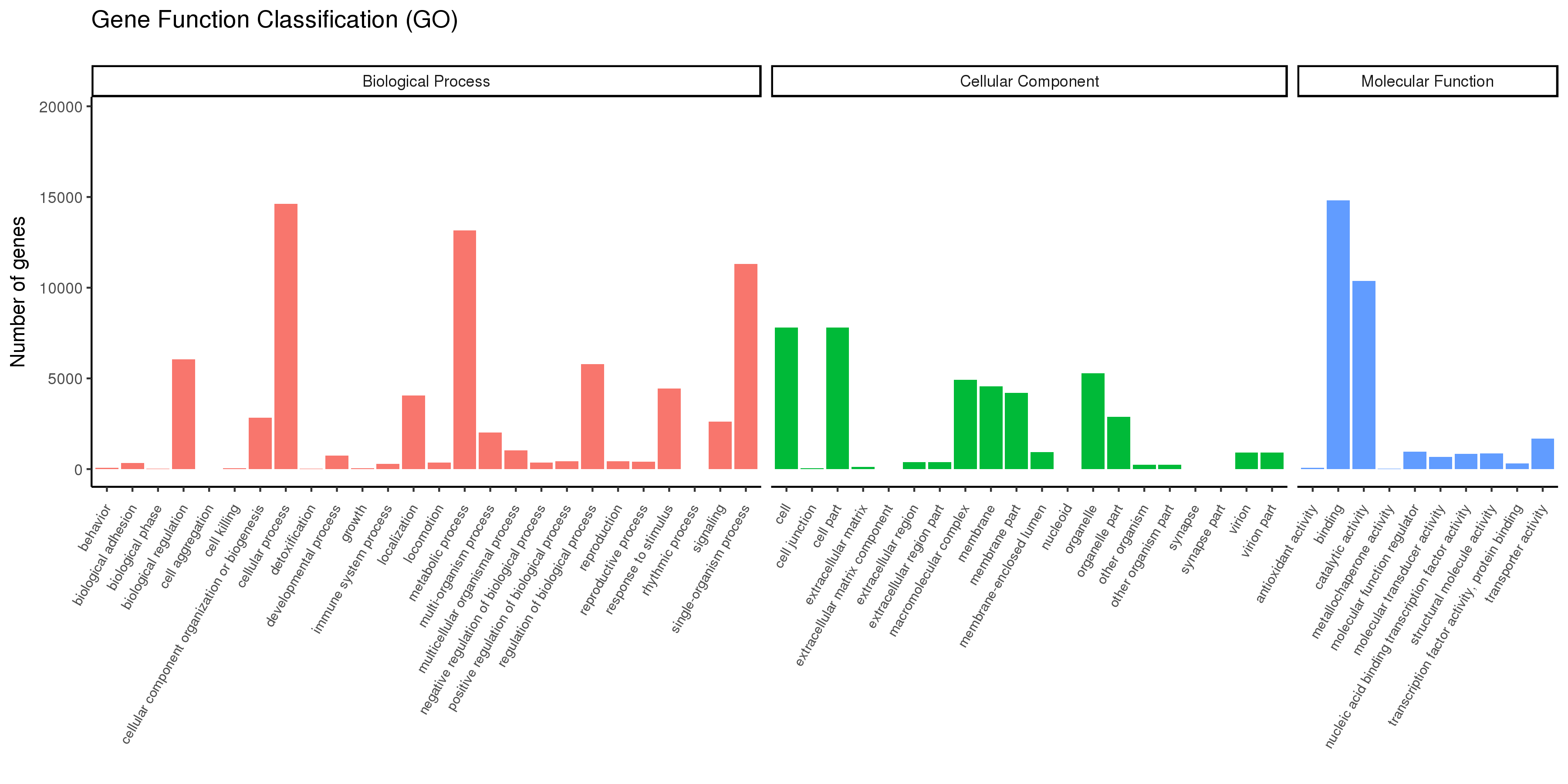

Supplement: Supplementary Figure S3 — Gene Ontology (GO) annotation of H. longicornis ovary genes. [file Image_3.PNG]

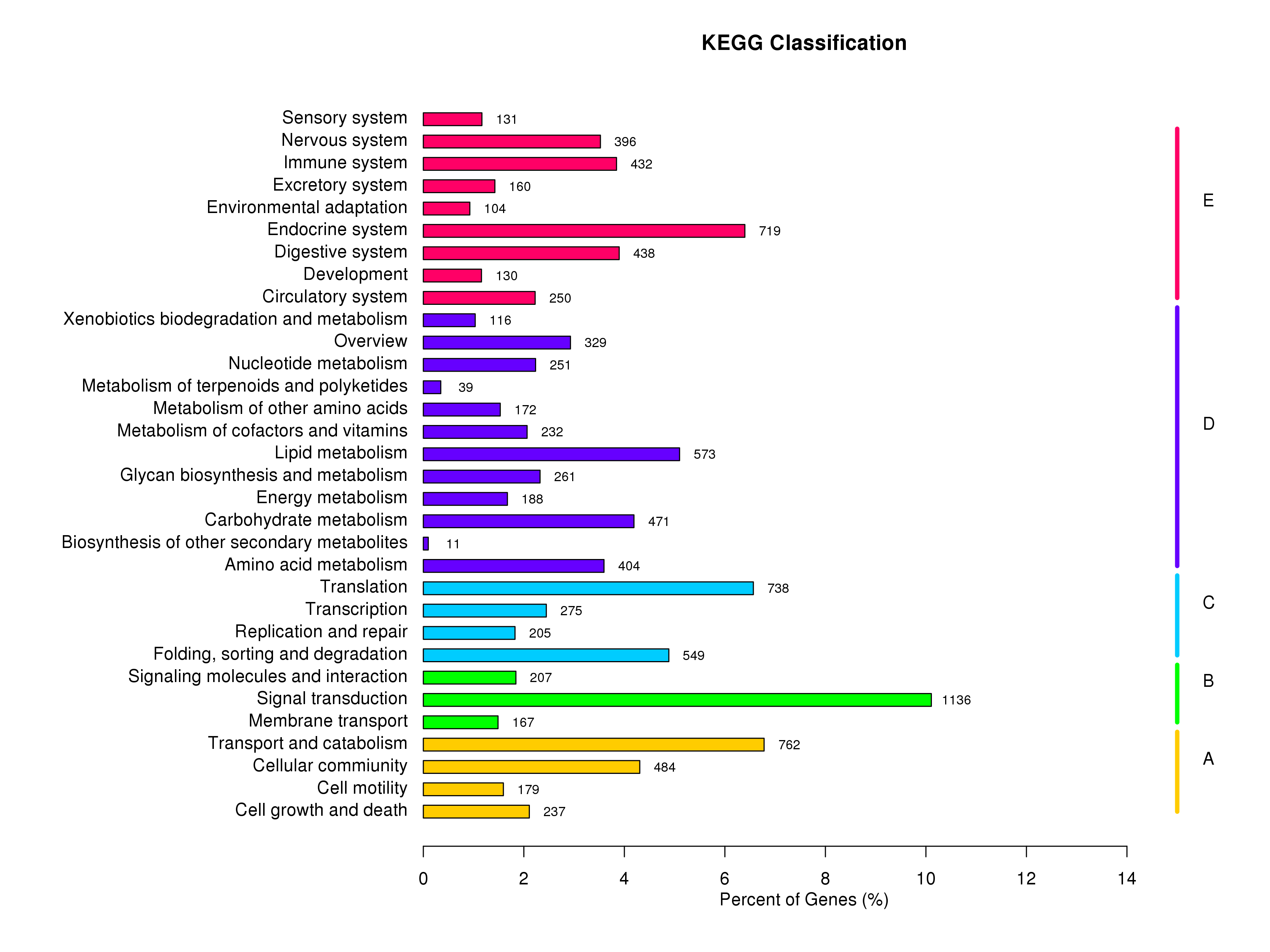

Supplement: Supplementary Figure S4 — The euKaryotic Ortholog Group (KOG) annotation of H. longicornis ovary genes. [file Image_4.PNG]

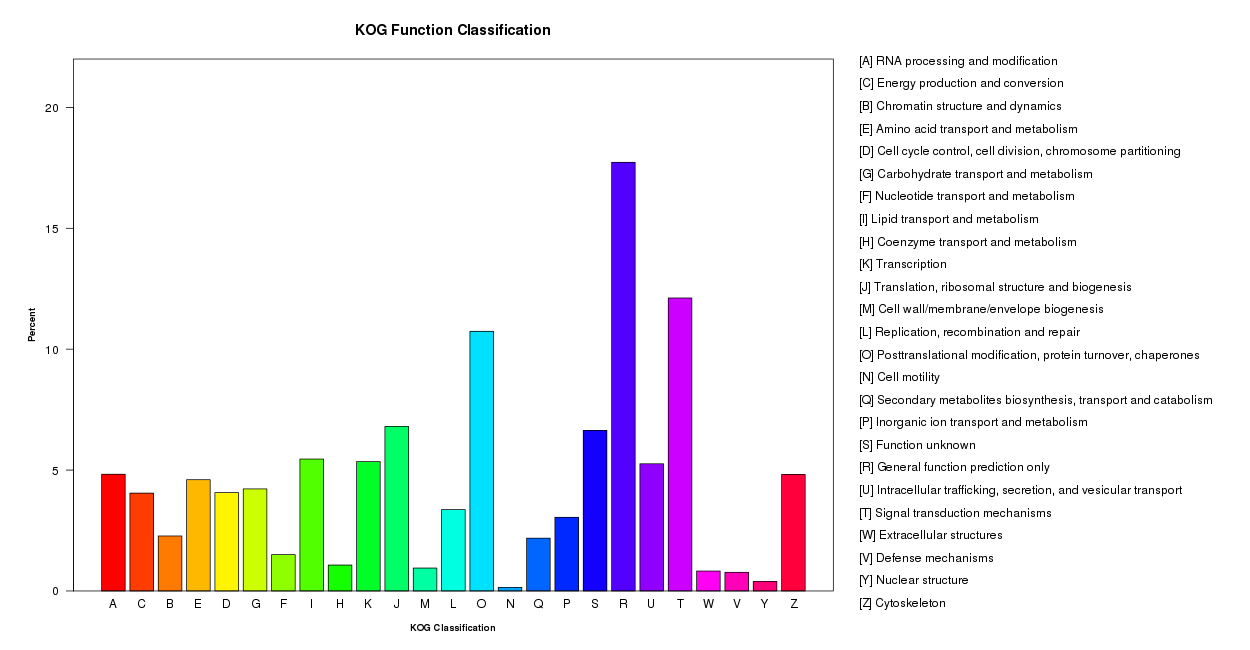

Supplement: Supplementary Figure S5 — Kyoto Encyclopedia of Genes and Genomes (KEGG) annotation of H. longicornis ovary genes. [file Image_5.PNG]

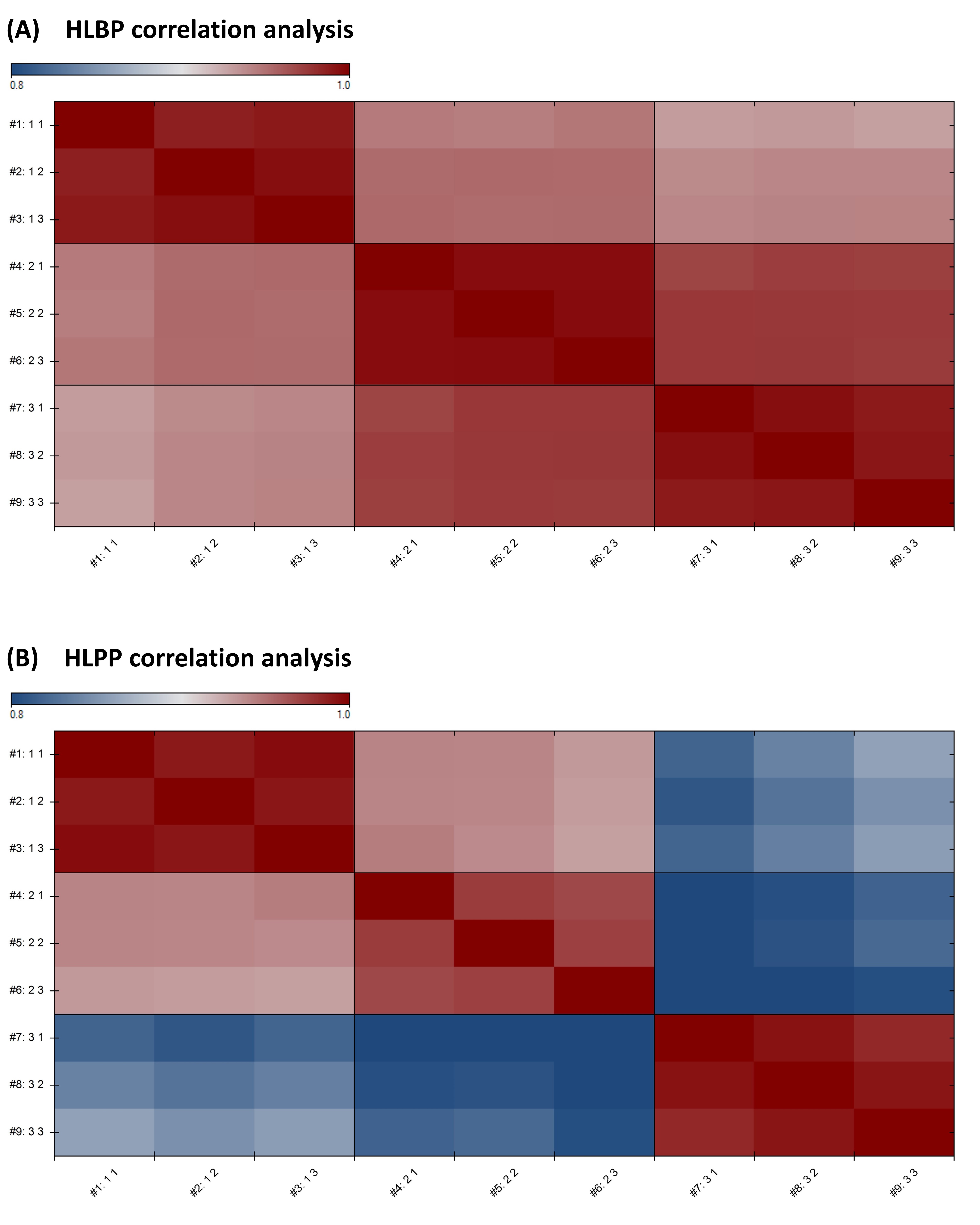

Supplement: Supplementary Figure S6 — Correlation analysis of mass spectrometry data in ovaries of the two reproductive groups of H. longicornis. [file Image_6.JPEG]

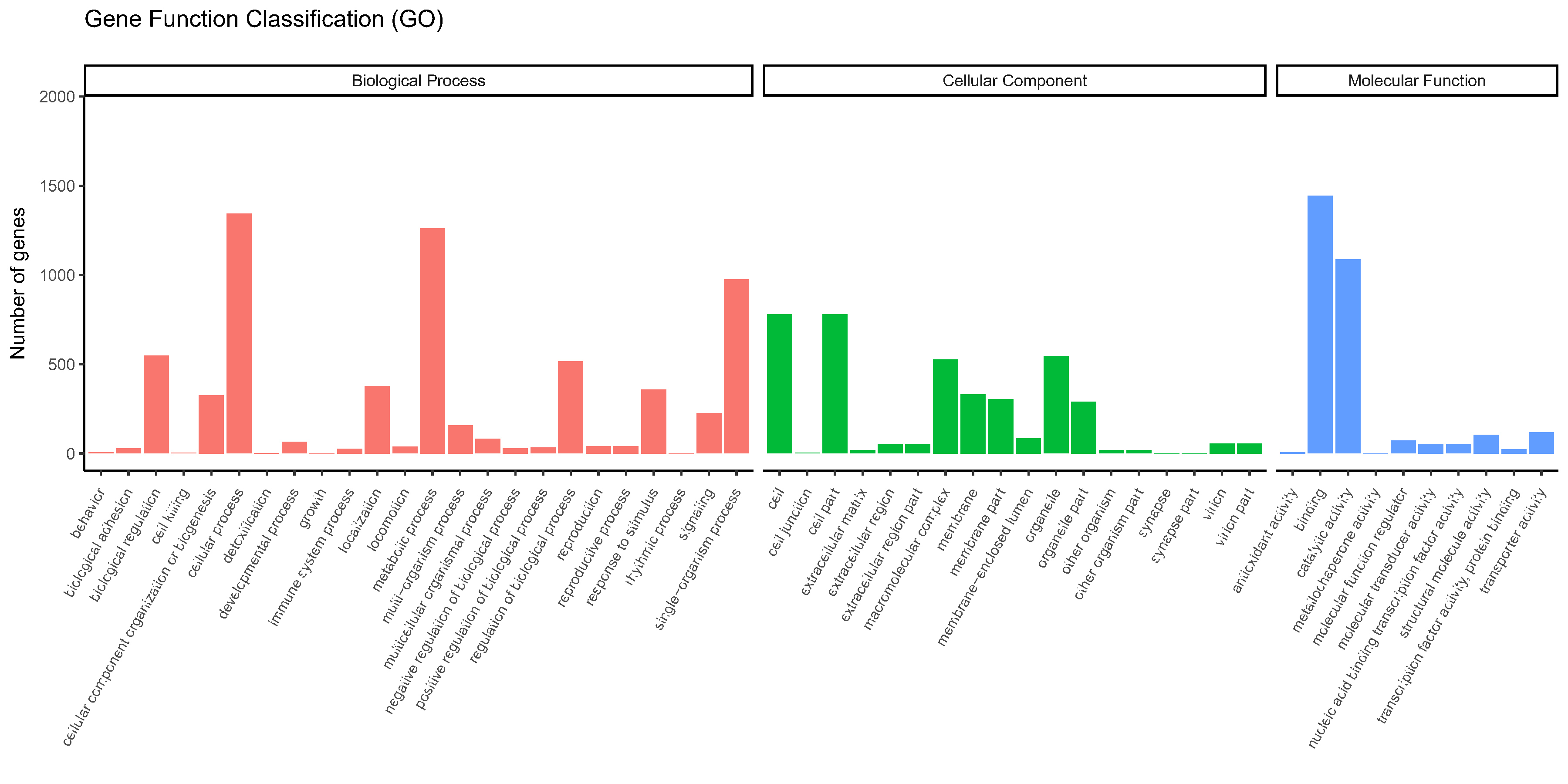

Supplement: Supplementary Figure S7 — Gene Ontology (GO) annotation of H. longicornis ovary proteins. [file Image_7.JPEG]

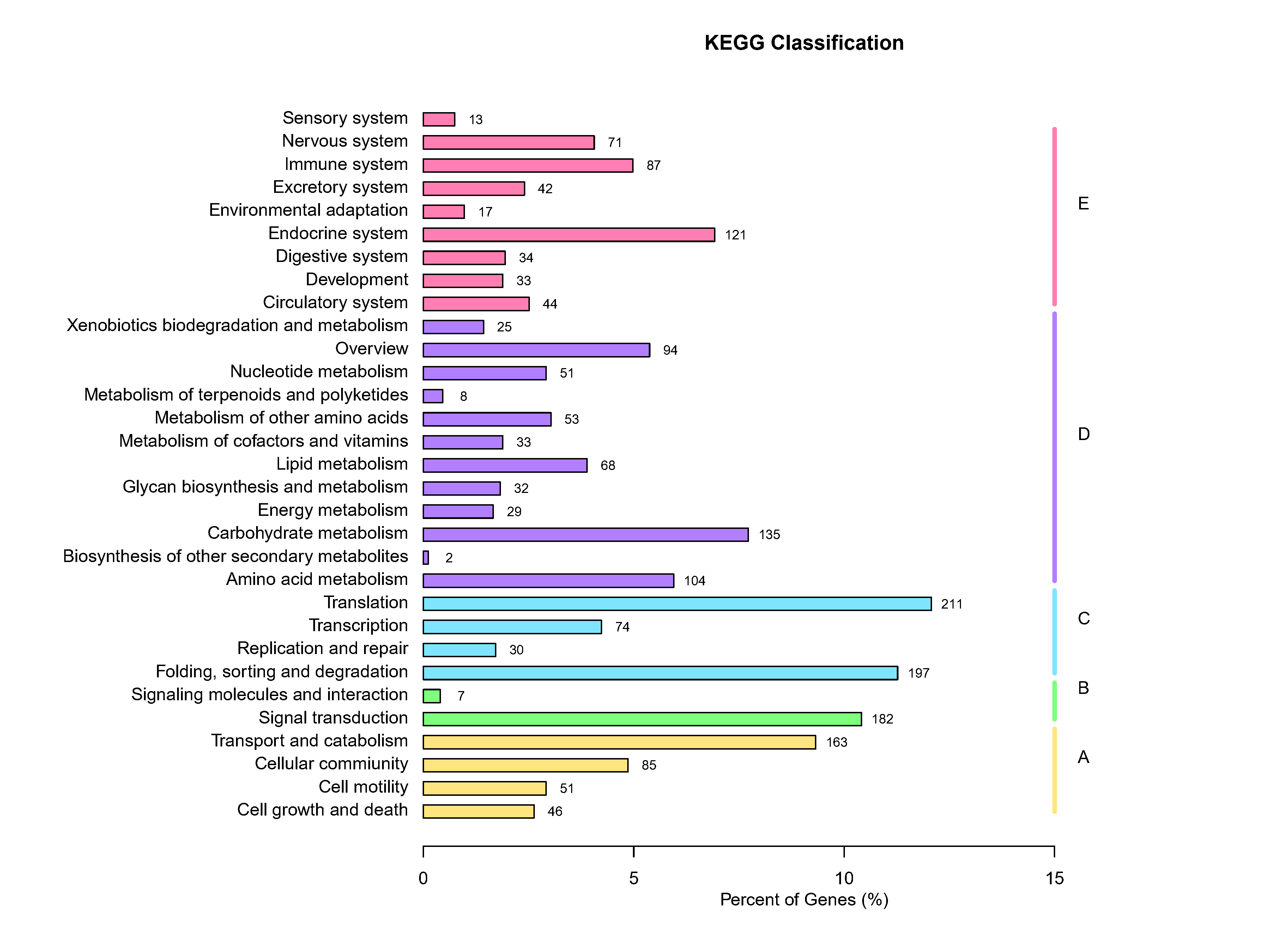

Supplement: Supplementary Figure S8 — The euKaryotic Ortholog Group (KOG) annotation of H. longicornis ovary proteins. [file Image_8.JPEG]

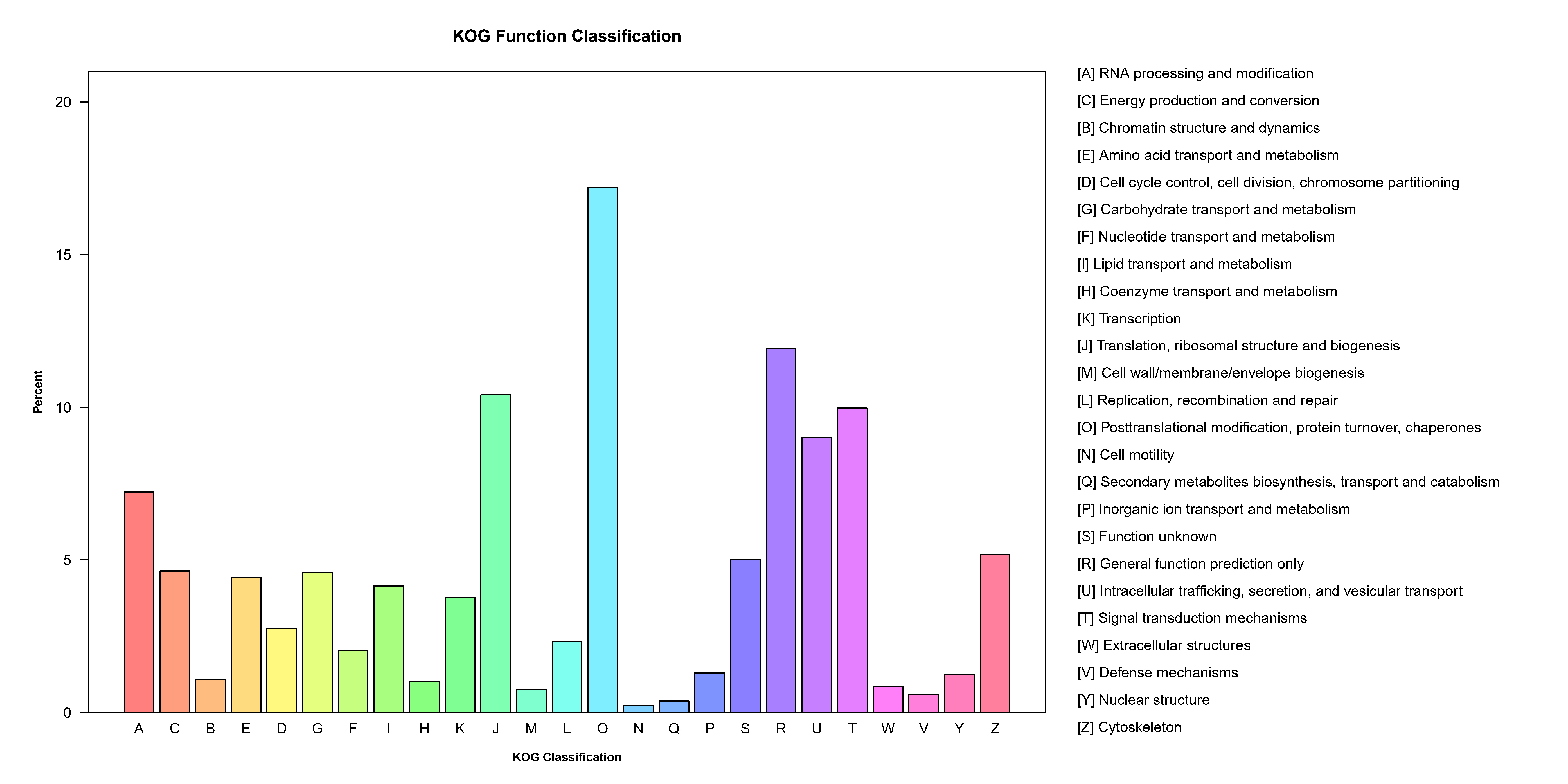

Supplement: Supplementary Figure S9 — Kyoto Encyclopedia of Genes and Genomes (KEGG) annotation of H. longicornis ovary proteins. [file Image_9.JPEG]
