# Supplementary material for: The Ovarian Development Genes of Bisexual and Parthenogenetic Haemaphysalis longicornis Evaluated by Transcriptomics and Proteomics
Source: Front Vet Sci. 2021 Dec 15;8:783404. doi: 10.3389/fvets.2021.783404 (PMC8714755; doi:10.3389/fvets.2021.783404)
Supplement: Supplementary Table S1 — Primer information of quantitative PCR genes. [file Table_1.docx]

**Table S1.** Primer information of quantitative PCR genes.

| Gene number | Primer sequence (F) | Primer sequence (R) | Product (bp) |
| --- | --- | --- | --- |
| HLBP-PP-T1 (16082.0) | ACCTGGTCATGGAGTCGGATCTG | AGCCTCGTGGCGTTGATGGA | 130 |
| HLBP-PP-T2 (19607.33672) | CCAGCCACGAGTCATTCCAGTTC | TGTTCTTAACGAGCCAGTAGTCCTTG | 138 |
| HLBP-PP-T3 (19607.38572) | ATCTTCAAAGGCGAGTTCATC | AAGTCCTTGGTGTTCTTGTTC | 152 |
| HLBP-UP1 (24273.0) | CGAGCCTGGAGGAAGTCTGGAT | CGTCGAAGGTGGTGATGAGGTTATC | 198 |
| HLBP-UP2 (19607.44128) | ACAACGCCGTGTACGCCATATC | TCCGATGAACTCCAGCGACTTGA | 149 |
| HLBP-UP3 (19607.47249) | CATAAGGATCGGAAGTGACTGGAAGG | CCATGTGCGGCTGATGAGGTT | 151 |
| HLBP-DOWN1 (19607.24316) | GGATGATCGCTGCCGACATACTG | ACGGAACACTTCGCTGCCACT | 137 |
| HLBP-DOWN2 (19607.34970) | CGGTGGAGAAGGTCTTCATCATTCG | GCTGCTGGTATGGCTGTGGGTA | 156 |
| HLBP-DOWN3 (19607.31271) | CCTTCCGCCTCATCACCTCTGA | CTCGTCCACCTGAGTGCCTTGTA | 167 |
| HLPP-UP1 (10378.0) | TGAATGTGGAAGAAGTTGGTGGTGAG | CAGCATGTGCAGAGGACAGGTC | 108 |
| HLPP-UP2 (19607.29323) | TCTTGATCTGCTTCTCCCTCGTGAA | TTCTCCACCGTGTCCTTGTCCTC | 151 |
| HLPP-UP3 (19607.26478) | CAAGCATGAGGAGAAGTCCGAGAAC | ATTGTGAGCACGCCGTCCTTG | 126 |
| HLPP-DOWN1 (19607.48479) | GGAAGGGAATCTGGCAGTTCAACAT | CAAGCAGTTCGTGGTCGTGGAA | 155 |
| HLPP-DOWN2 (22462.0) | GGAACAACCTGAACGAGCAAGTGA | CGGCCATCTGCATGTAGACCAAG | 123 |
| HLPP-DOWN3 (19607.34957) | CTACGGCAAGGCTGGCATCAAC | TTTGGCGTGCGTCGAGTCCT | 85 |
