# Supplementary material for: The Ovarian Development Genes of Bisexual and Parthenogenetic Haemaphysalis longicornis Evaluated by Transcriptomics and Proteomics
Source: Front Vet Sci. 2021 Dec 15;8:783404. doi: 10.3389/fvets.2021.783404 (PMC8714755; doi:10.3389/fvets.2021.783404)
Supplement: Supplementary Table S2 — Data acquisition and assembly results of the transcriptome data and proteome. [file Table_2.docx]

**Table S2.** Data acquisition and assembly results of the transcriptome data and proteome.

| **Sample** | **Transcriptome data** | | | | | | |  | **Proteome data** | | | | | | | |
| --- | --- | --- | --- | --- | --- | --- | --- | --- | --- | --- | --- | --- | --- | --- | --- | --- |
|  | **Raw Rengorgdds** | **Clengorgdn rengorgdds** | **Clengorgdn bases** | **Error（%）** | **Q20（%）** | **Q30（%）** | **GC（%）** |  | **Precursors** | **Modified Peptides** | **Peptides** | **Protein Groups** | **Proteins** | **Pengorgdk Capacity** | **Median FWHM** | **Median Pengorgdk Width** |
| HLBP-early-1 | 70003372 | 68452894 | 10.27G | 0.03 | 97.35 | 92.99 | 56.74 |  | 13826 | 11393 | 11273 | 1982 | 2017 | 218.48 | 0.324 | 0.549 |
| HLBP-early-2 | 65776374 | 62967698 | 9.45G | 0.03 | 97.29 | 92.84 | 57.13 |  | 14668 | 12120 | 11983 | 2041 | 2079 | 230 | 0.3077 | 0.5215 |
| HLBP-early-3 | 74958828 | 72926666 | 10.94G | 0.03 | 96.87 | 91.94 | 57 |  | 14879 | 12298 | 12162 | 2045 | 2082 | 226.81 | 0.312 | 0.5288 |
| HLBP-partially-1 | 60083766 | 58340170 | 8.75G | 0.03 | 97.67 | 93.62 | 55.31 |  | 14985 | 12364 | 12211 | 2036 | 2073 | 232.65 | 0.3042 | 0.5155 |
| HLBP-partially-2 | 67524152 | 65534748 | 9.83G | 0.03 | 96.73 | 91.59 | 52.69 |  | 15022 | 12394 | 12240 | 2053 | 2090 | 231.23 | 0.306 | 0.5187 |
| HLBP-partially-3 | 50047248 | 48916418 | 7.34G | 0.03 | 97.28 | 92.68 | 55.4 |  | 15086 | 12443 | 12288 | 2052 | 2087 | 227.3 | 0.3113 | 0.5277 |
| HLBP-engorgd-1 | 66099272 | 64721570 | 9.71G | 0.03 | 97.03 | 92.31 | 57.5 |  | 15399 | 12733 | 12570 | 2075 | 2113 | 237.83 | 0.2975 | 0.5042 |
| HLBP-engorgd-2 | 57372376 | 56337204 | 8.45G | 0.03 | 97.34 | 92.94 | 57.08 |  | 15212 | 12595 | 12440 | 2063 | 2098 | 242.44 | 0.2918 | 0.4946 |
| HLBP-engorgd-3 | 51024938 | 49726792 | 7.46G | 0.03 | 95.01 | 88.11 | 57 |  | 15258 | 12637 | 12481 | 2068 | 2104 | 241.69 | 0.2928 | 0.4963 |
| HLPP-early-1 | 74678146 | 72804838 | 10.92G | 0.03 | 97.69 | 93.72 | 56.87 |  | 14534 | 12000 | 11865 | 1996 | 2033 | 229.4 | 0.3084 | 0.5227 |
| HLPP-early-2 | 77396130 | 75339152 | 11.3G | 0.03 | 97.49 | 93.28 | 57.31 |  | 14735 | 12189 | 12045 | 2031 | 2068 | 237.31 | 0.2981 | 0.5053 |
| HLPP-early-3 | 61026890 | 58390140 | 8.76G | 0.03 | 97.35 | 93.01 | 56.82 |  | 14631 | 12087 | 11943 | 2019 | 2055 | 237.19 | 0.2983 | 0.5056 |
| HLPP-partially-1 | 49439084 | 48349968 | 7.25G | 0.03 | 97 | 92.1 | 53.49 |  | 13974 | 11714 | 11578 | 1970 | 2000 | 307.74 | 0.2299 | 0.3897 |
| HLPP-partially-2 | 52441704 | 51020272 | 7.65G | 0.03 | 95.4 | 88.77 | 53.16 |  | 13281 | 11150 | 11024 | 1922 | 1952 | 314 | 0.2253 | 0.3819 |
| HLPP-partially-3 | 45930236 | 44742344 | 6.71G | 0.03 | 97.11 | 92.3 | 55.47 |  | 14264 | 11938 | 11795 | 1996 | 2032 | 334.66 | 0.2114 | 0.3583 |
| HLPP-engorgd-1 | 50835954 | 49794324 | 7.47G | 0.03 | 95.46 | 88.96 | 55.15 |  | 12945 | 10748 | 10638 | 1871 | 1902 | 227 | 0.3117 | 0.5284 |
| HLPP-engorgd-2 | 46868018 | 45535370 | 6.83G | 0.03 | 95.49 | 89 | 55.06 |  | 12920 | 10710 | 10595 | 1877 | 1908 | 226.39 | 0.3126 | 0.5298 |
| HLPP-engorgd-3 | 60633690 | 59472228 | 8.92G | 0.03 | 95.62 | 89.23 | 55.96 |  | 13041 | 10824 | 10706 | 1874 | 1908 | 220.58 | 0.3208 | 0.5437 |
